# Quantum Dots for Cancer-Related miRNA Monitoring

**DOI:** 10.1021/acssensors.2c00149

**Published:** 2022-04-29

**Authors:** Catarina S. M. Martins, Alec P. LaGrow, João A. V. Prior

**Affiliations:** † International Iberian Nanotechnology Laboratory, 4715-330 Braga, Portugal; ‡ LAQV, REQUIMTE, Laboratory of Applied Chemistry, Department of Chemical Sciences, Faculty of Pharmacy, University of Porto, Rua de Jorge Viterbo Ferreira, No. 228, 4050-313 Porto, Portugal

**Keywords:** bioconjugation, biosensor, bioimaging, cancer, detection, functionalization, miRNA, monitoring, quantum dots

## Abstract

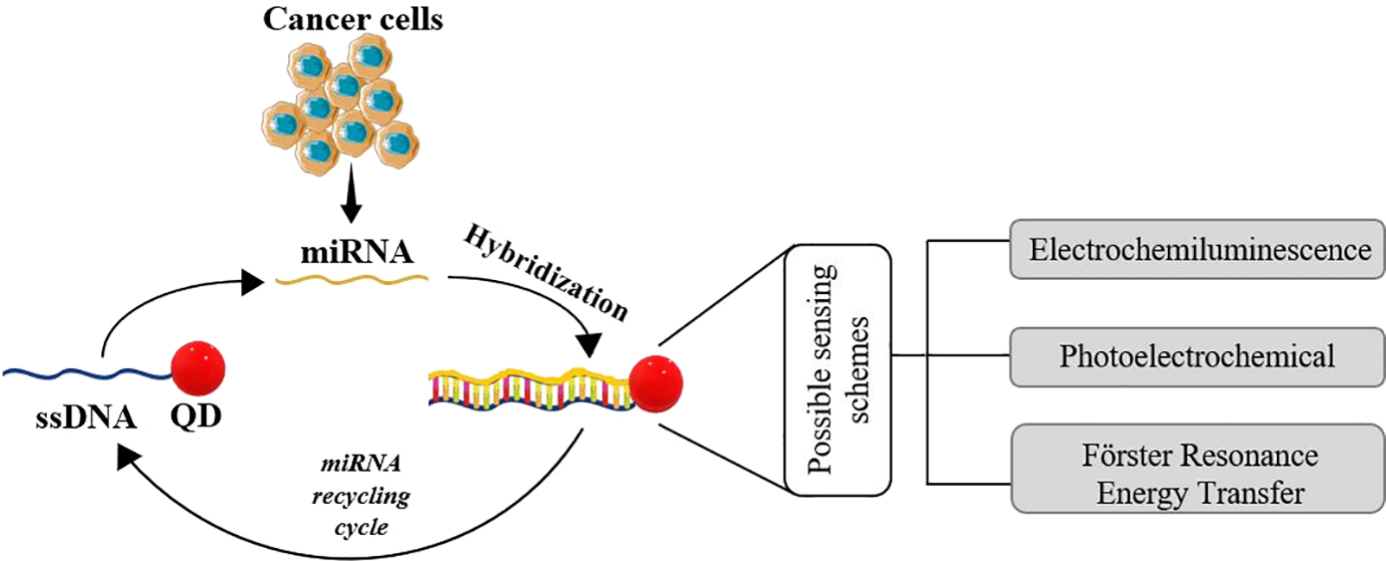

Quantum dots (QDs) possess exceptional optoelectronic properties that enable their use in the most diverse applications, namely, in the medical field. The prevalence of cancer has increased and has been considered the major cause of death worldwide. Thus, there has been a great demand for new methodologies for diagnosing and monitoring cancer in cells to provide an earlier prognosis of the disease and contribute to the effectiveness of treatment. Several molecules in the human body can be considered relevant as cancer markers. Studies published over recent years have revealed that micro ribonucleic acids (miRNAs) play a crucial role in this pathology, since they are responsible for some physiological processes of the cell cycle and, most important, they are overexpressed in cancer cells. Thus, the analytical sensing of miRNA has gained importance to provide monitoring during cancer treatment, allowing the evaluation of the disease’s evolution. Recent methodologies based on nanochemistry use fluorescent quantum dots for sensing of the miRNA. Combining the unique characteristics of QDs, namely, their fluorescence capacity, and the fact that miRNA presents an aberrant expression in cancer cells, the researchers created diverse strategies for miRNA monitoring. This review aims to present an overview of the recent use of QDs as biosensors in miRNA detection, also highlighting some tutorial descriptions of the synthesis methods of QDs, possible surface modification, and functionalization approaches.

Over the past few years, death from cancer has grown to such an extent that in late 2020 it has been considered the major cause of death worldwide, with almost 10 million recorded deaths.^1^ Success in cancer treatment is highly dependent on the stage it is detected, so an early diagnosis is imperative for effective treatment.^2^ Aiming at early diagnosis, scientists are actively looking for something effective in this fight against cancer, creating a great demand for new approaches for cancer detection and monitoring in live cells. Currently, in the hospitals, tissue biopsies are used as a diagnostic procedure, although it is a laborious and invasive procedure.^3^

To circumvent the use of this invasive procedure, researchers have been searching for cancer biomarkers that allow for an early diagnosis of the disease. A biomarker can be a protein, a small fragment of a protein, or a deoxyribonucleic acid (DNA)- or ribonucleic acid (RNA)-based structure. Usually, when these biomarkers are abnormally expressed in a tissue, it is indicative of a disease and representative of a specific stage of disease, revealing an alteration in a cellular process. Their detection and monitoring provide the diagnostic and further treatment of the associated disease.^4^

Recent evidence has shown that RNA species that are part of extracellular vesicles (EVs) cargo, have an important role in the tumor microenvironment (TME) modulation and cancer progression.^5^ The TME is composed by diverse cells in the vicinity of the tumor, such as fibroblast, endothelial, immune, fat, neural, epithelial, and mesenchymal stem cells, as well as the communication networks established between them, which include soluble and insoluble factors, EV, and the extracellular matrix. Among the noncoding RNAs (ncRNAs), the micro RNAs (miRNAs)^6^ and long noncoding RNAs (lncRNAs)^7^ are the two most widely studied biomarkers due to their impact on post-transcriptional gene regulation.^8^ miRNAs are usually composed by 19–23 nucleotides and perform regulatory functions and play a critical role in cancer’s pathology, because they are involved in gene expression control and have deregulated levels during cancer progression.^9,10^ Twenty-eight years ago, Lee et al.^11^ discovered the first miRNA in the nematode *Caenorhabditis elegans*, lin-4, and only then the importance of miRNAs began to be revealed. miRNAs are responsible for some physiological cell cycle processes, such as differentiation, proliferation, and apoptosis,^12^ and, also, hematopoietic differentiation, tumor metastasis, among others.^13^ However, in most kinds of tumors, miRNA is upregulated, and it can act like a post-transcriptional repressor.^14^ Considering the emerging roles of miRNA, it can be considered an essential cancer biomarker and its clinical monitoring is of prime importance.^15^ Among all of the already identified and well-known miRNA, miRNA-21 is the most common overexpressed miRNA in different types of cancer, namely, glioblastoma, breast, and gastrointestinal cancer.^10^

In the literature, several analytical methods for the monitoring of miRNA can be found, namely, real-time quantitative polymerase chain reaction (qRT-PCR), electrochemical sensing, Northern blotting, and DNA microarrays. These conventional techniques, despite their high sensitivity, good performance, and specificity, have some limitations, because they involve high-cost equipment, long processing time, and specialized personnel.^13,16^ For in vivo detection, the techniques and materials used must be biocompatible and of very low dimensions, at small-molecular or atomic level, making the nanomaterials highly attractive for the development of new monitoring schemes of miRNAs in living cells. The new monitoring schemes must rely on the measurement of an analytical signal easily to detect without the need of high-end equipment. Thus, fluorescent nanomaterials exploited together with microscopy techniques constitute a promising choice for monitoring cancer miRNAs biomarkers.

These nanomaterials, explored by the researchers, are semiconductor colloidal nanocrystals, namely, quantum dots (QDs), of quasi-zero dimensions and natively fluorescent, with broad excitation spectra and narrow emission peaks, these being size-dependent.^17^ The “quantum confinement” effect, represented in Figure 1, is a particularity of QDs that depends on particle size and on the Bohr radius of the material. The confinement is a way to explain the fluorescence emitted by the quantum dots, being responsible for the exquisite optoelectronic properties they possess.^18,19^ The smaller the particle size, the larger the exciton Bohr radius, leading to the confinement effect since the band gap energy between the valence band and the conduction band increases.^20,21^ Due to this phenomenon, the color of QDs can be tuned, by only varying their size.^22^

**Figure 1:**
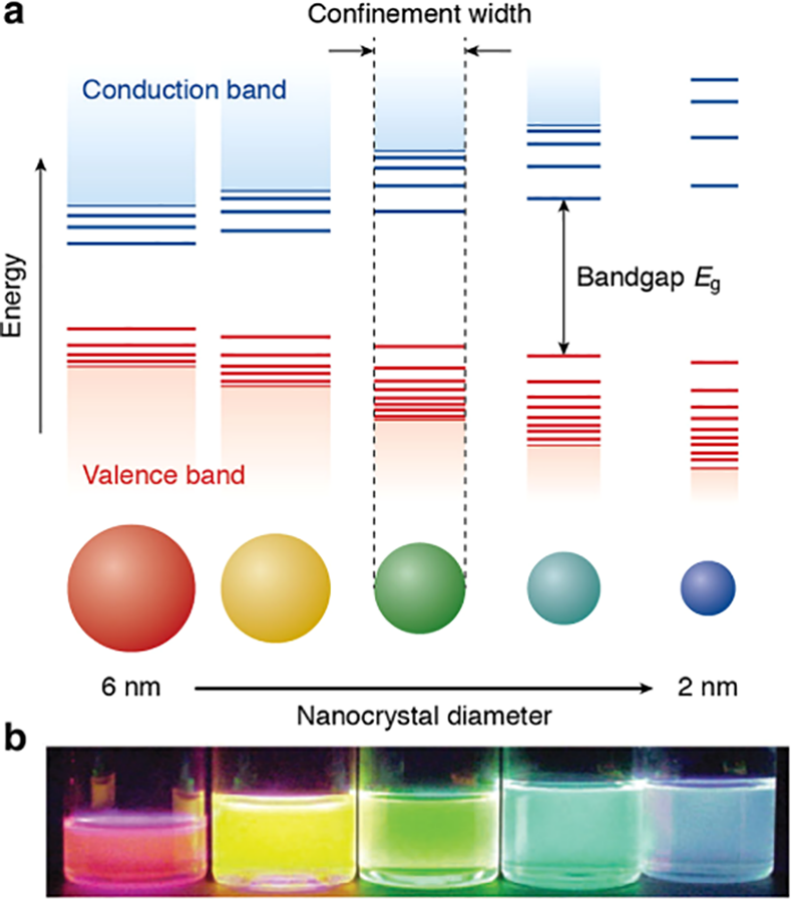
(a) Representative scheme of “quantum confinement” effect indicating that smaller QDs have higher band gap energy levels; (b) different colors of five colloidal dispersions of CdSe QDs with diameters from 6 to 2 nm, after UV excitation. Reprinted with permission from ref (23), Copyright 2016 The Authors under Creative Commons 4.0 Attribution License, published by Springer Nature.

QDs are easy to functionalize and are generally photostable, resistant to photobleaching, which makes them attractive for being widely used.^17^ They are constructed from semiconductor materials, usually composed of a core that determines the optical features and a shell to increase the quantum yield (QY). The most fabricated QDs are composed of semiconductors materials, from the groups IIB–VIB from the periodic table, such as Cd-based chalcogenide (S, Se, Te), represented by homogeneous structures, such as CdTe or CdSe,^24^ core–shell structures, namely, CdTe/CdS, and ternary structures, such as CdTeSe,^25−27^ but they also can be composed by silicon^28^ and germanium^29^ and by the elements from groups IIIB–VB (e.g., InP and InAs) or IVB–VIB from the periodic table.^24^

Considering the mentioned advantages of QDs, namely, nucleic acids-functionalized QDs, as working biosensing-based materials, several detection schemes have been proposed using QDs as fluorescence probes for the detection of miRNAs. Some reviews about miRNAs and their interaction with nanomaterials can be found in the scientific literature. Yet, some of the published articles are focused only on specific nanoparticles other than QDs or on a specific type of cancer, hence limited specific biomarkers.^30−36^ Other reviews focus the discussion on works involving not only cancer-related miRNAs but also nanomaterials of different chemical nature, containing a reduced number of analyzed original articles.^3,37−40^

The present work describes the main fundamentals of quantum dots, their synthesis processes, surface modifications, and subsequent functionalization. The use of quantum dots as sensing probes for cancer-related miRNA and other biomarkers are thoroughly discussed herein. Additionally, a final section presenting the exploitation of QDs for bioimaging of cancer cells is also included, as the imaging mechanism relied on the interaction between nucleic acid-functionalized QDs and specific cancer biomarkers. The present work aims at emphasizing the potential of QDs functionalized with specific sequences of nucleic acids.

## Quantum Dots

### Synthesis Methods

Quantum dots can be synthesized by several routes (chemical, physical, or biological) organized in two main categories, namely, the top-down and the bottom-up approaches. The top-down procedures consist of thinning a bulk material into small fragments, and in an opposite way, the bottom-up approaches are based on the growth of particles from a precursor, synthesized through nucleation and growth processes, to form stable structures at the atomic level.^41,42^ The most common top-down technique is lithography, which can be divided into optical, electron beam, soft, nanoimprint, scanning probe, or block copolymer lithography. Nevertheless, other procedures can be used, such as laser ablation, electrochemical etching, and liquid-phase exfoliation.^43^ These top-down techniques present some disadvantages, such as limited control of the particles’ final size and shape and low yield, among others. Therefore, these methods are less commonly used for the QDs synthesis.

Bottom-up approaches include hydrothermal/solvothermal methods, microwave irradiation, and soft-template, pyrolysis, and wet-chemical reactions to produce quantum dots.^42,44^ Other approaches described in the literature are atomic layer deposition, sol–gel nanofabrication, molecular self-assembly, and vapor-phase deposition.^43^ Since these approaches allow for precise control of the size and the morphology of single crystalline quantum dots, they are often the first choice of the authors to synthesize QDs.^42^ The hydrothermal/solvothermal methods are based on the reaction between the precursors present in a reaction vessel: when the main solvent is water, the approach is named hydrothermal, and when the process involves the use of an organic solvent, the approach is called solvothermal. Generally, the hydrothermal approach is typically selected to synthesize biocompatible QDs, using, for example, a three-neck round-bottom glass flask, with conventional heating (electrically powered isomantles or oil baths) and a condensing system. In the hydrothermal approach, the synthesis can be performed in a broad spectrum of escalating temperatures, from smaller to very high values (50–380 °C), under moderate to high pressure.^45^ An example of the hydrothermal method was exploited by Yuan et al.^49^ to synthesize carboxyl-modified CdTe QDs (in aqueous medium) to sensitize the photoactive nanomaterials of Bi_2_Te_3_ nanosheets, allowing the assembly of a photoelectrochemical biosensor. The goal of the as-synthesized biosensor was the detection of miRNA-21 on lysates of HeLa and MCF-7 cells. The cadmium precursor was mixed with the capping agent, mercaptopropionic acid (MPA), followed by a pH adjustment to 12 with 1 M NaOH and deoxygenation with N_2_ airflow. The reagents NaBH_4_ and Na_2_TeO_3_ were added under stirring into a three-necked flask and refluxed at 100 °C for 6 h, originating QDs of about 5 nm in diameter. Also, Lv et al.^50^ synthesized MPA-capped CdTe QDs to constitute a fluorescence probe for miRNA-122 detection, after the functionalization of the QDs with single-stranded DNA (ssDNA) complementary to the analyte. The preparation of the aqueous QDs was made through several steps. First, in one three-neck round-bottom glass flask, tellurium powder was dissolved in deoxygenized ultrapure water, under stirring, followed by the addition of NaBH_4_ to prepare the NaHTe solution. In another three-neck round-bottom glass flask equipped with a flux condenser, the cadmium precursor was dissolved in ultrapure water with constant N_2_ airflow, and the capping agent was added. The pH was adjusted to 10 with 1 M NaOH solution. After 30 min of constant stirring at room temperature, the previously prepared NaHTe solution was injected into the second three-neck round-bottom glass flask, maintaining the N_2_ airflow, and refluxed at 100 °C for 2.5 h. The obtained MPA-capped CdTe QDs were 2.23 nm in size, emitted fluorescence at 535 nm, and were ready to be functionalized with DNA and to be used as a probe.

Microwave-assisted synthesis while providing fast heating rates, and at the same time uniform heating with high-energy efficiency, allows for shorter synthesis times, reducing the required time from hours to minutes. Another advantage of this method is the ability to easily manage each particles’ size distribution due to the capacity of the microwave to quickly attain the required temperature, both in the necessary heating process, as well as the cooling of the mixture at the end of the reaction. This aspect is of paramount significance considering the QDs fluorescence emission profile is dependent on the nanoparticles’ size^47^ as rapid heating rates will focus the nucleation event into a burst nucleation and rapid cooling can limit Ostwald ripening processes that increase the particle size. In sum, it can be considered an environment-friendly methodology since it requires less energy, than the conventional methods, to control the heating process during the synthesis; also, the entire procedure occurs in a shorter period of time.^51,52^ A microwave irradiation-based synthesis of CdTe/CdS core–shell QDs was conducted by Su et al.^13^ to produce a nanoprobe miRNA-21 detection. The authors proposed a synthesis in an aqueous solution and used MPA as a capping agent. In a first step, they synthesized CdTe QDs emitting at 520 nm, through microwave irradiation during 1 min at 100 °C, upon the mixture of the precursors and the stabilizing agent in a reaction vessel. After the purification of the as-synthesized CdTe QDs (core), the precursors of the CdS shell were added to the solution and subjected again to microwave irradiation at 120 °C for several minutes, allowing in this way the growth of the shell on the surface of previously synthesized CdTe QDs. The as-prepared CdTe/CdS QDs showed a spherical shape and an average diameter of 3 nm. Next, the CdTe/CdS QDs were functionalized with thiolated ssDNA by ligand-exchange approach, with an emission peak at 595 nm.

Another advantage of the bottom-up processes is that they produce nanomaterials with ligands natively on their surface. This is an advantage, since one can choose ligands to increase the biocompatibility of the QDs and to add to their surface specific chemical groups for posterior bioconjugation with specific functional molecules such as nucleic acid sequences, enabling the development of base nanomaterials for specific and sensitive QDs-based sensors. These ligands can further be easily modified through several approaches, which will be explored in the next section. It should be noted that when solvothermal approaches are used, additional steps are needed to exchange or engulf the ligands native to the surface to allow for further modifications; this makes these approaches less desirable for water-based applications.

### Surface Modifications Approaches

Currently, live cell imaging, bioanalytical assays, imaging techniques, tracking of particles or molecules of interest, and energy-transfer-based sensing probes are some of the multiple applications QDs can have in the biomedical field.^54^ Since most quantum dots have a heavy metal core (such as cadmium), there are some concerns about their use, essentially, for biomedical purposes, due to the potential toxicity.^55^ The QDs’ toxicity can be inferred by one or the combination of the following mechanisms: the disintegration of the nanoparticles with the consequent release of free heavy metal ions (such as Cd^2+^) or the generation of reactive oxygen species.^56^ The ratio of toxic cadmium can be reduced by doping QDs^57^ or even through the addition of a proper shell around the core (core–shell QDs), since the nature of the surface coating is intimately correlated with the toxicity of the QDs, which prevents oxidation of the core and the possible release of free heavy metal ions.^42^ However, to circumvent the toxicity of cadmium-based quantum dots, silver-, indium-, carbon-,^58^ or silicon-containing QDs can be used, since the in vitro cytotoxicity induced by QDs can be dependent on a single factor or a combination of several factors, such as the type of the core, the dose, the shape and the size, and the surface chemistry, as well as the type of cell.^59^ Nevertheless, many modifications can be made to the QDs’ surface to circumvent this problem and to enhance the quantum dots’ biocompatibility to be used in the above-mentioned applications, without interfering with vital functional processes of the living test objects.^60^ For example, it is frequently the addition of a zinc sulfide (ZnS) shell to a cadmium-based core, not only to reduce the toxicity of QDs but also to improve their biocompatibility and colloidal stability.^61^ Aiming at the reduction of the toxicity of CdTe QDs in biological environments, through the reduction of the release of free Cd^2+^ ions to the medium, Liu et al.^62^ synthesized core–shell CdTe/ZnS QDs. The biocompatibility of as-synthesized CdTe/ZnS QDs was evaluated by hemolysis assays and compared with TGA-capped CdTe QDs. As a result, the hemolysis caused by the core–shell nanoparticles was lower than the TGA-CdTe QDs, for the same concentration and time of exposure. Also, the toxicity due to the release of free heavy metal ions was analyzed by dialysis and quantified by atomic absorption spectroscopy, and the results showed that the amount of free released Cd^2+^ ions was lower in the core–shell QDs than in CdTe QDs. Accordingly with the scientific literature, CdTe/ZnS QDs are widely used as a probe for cancer-related miRNA detection.^12,63−65^

The replacement of hydrophobic ligands by water-soluble bifunctional molecules is a requirement to develop high-quality QDs for biomedical sensing devices.^22^ Some essential characteristics to make QDs ideal for bioanalytical applications are the stability in aqueous solvents over a broad range of pH and ionic strength, the maintenance of high photoluminescence efficiency, and a small nanoparticle diameter to facilitate the uptake by living cells.^42^

**Figure 2:**
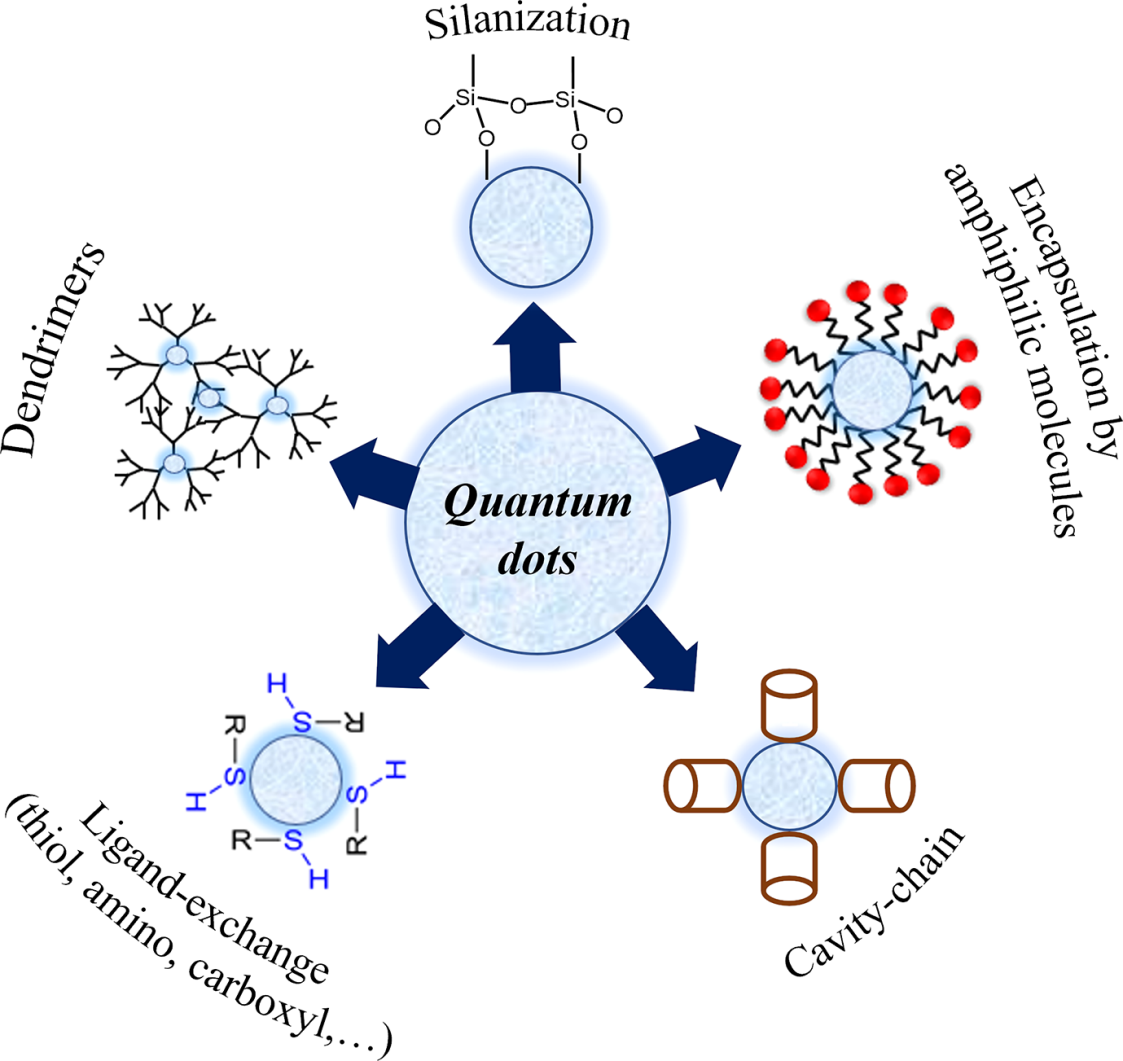
Schematic representation of quantum dots surface modifications strategies.

Figure 2 illustrates the most described surface modification strategies in the literature: ligand exchange through functional groups; silanization by coating the QDs surface with a silica-based (SiO_2_) shell; encapsulation by amphiphilic molecules, which allow the control of the QDs’ permeability; cavity–chain by using polymers with hydrophobic cavities; and dendrimer technology,^66−68^ which will be discussed throughout the next sections.

#### Ligand Exchange

The principle of the modification of the QDs’ surface through ligand exchange is based on the strength of interaction of the functional anchor groups (-SH, -NH_2_, or -COOH), which will be larger than the pre-existing ligands, in the external layer of the QDs. For example, when QDs are synthesized through solvothermal methods they contain a hydrophobic layer, composed by organic ligands such as trioctylphosphine (TOP), trioctylphosphine oxide (TOPO), hexadecyl amine (HDA), oleic acid (OA), or tetradecyl phosphonic acid (TDPA), which has to be replaced by bifunctional molecules, to enable bioconjugation.^67,69^ In this procedure, organic ligands can remain behind and might be toxic, which can be a disadvantage. Also, degradation or oxidation processes can occur during the exchange protocol. Oxidation during ligand exchange, either due to harsh chemicals used or due to exposure of the surface when the ligands are removed, can be an important limitation. Organic solvent syntheses might allow a better control of the produced nanomaterials, but the posterior ligand-exchange procedure can be a limiting step, whereas aqueous syntheses are an obvious candidate; however, these can present more challenges to obtaining proper size distributions, quantum yield, and narrow emission fluorescence.^17^

Jou et al.^63^ applied the ligand-exchange approach to prepare hydrophilic QDs for the sensing of miRNA-141. The authors acquired commercial CdSe/ZnS QDs capped with octadecylamine (ODA). These were precipitated with methanol and afterward resuspended in chloroform. Then, by exploiting the ligand-exchange procedure, the ODA hydrophobic capping was replaced by l-glutathione reduced (GSH), by adding GSH in methanol and mixing at 50 °C for 2 h. After cooling to 27 °C, the mixture was allowed to react overnight. The final addition of an aqueous solution of NaOH allowed GSH-capped QDs to be separated to the aqueous phase.

Thiol ligands, such as GSH, MPA, mercaptoacetic acid (MAA), etc., are often selected to exchange the hydrophobic ligands on the surface of QDs. The thiol functional group provides the link to the metal element of QDs, while other functional groups such as carboxyl groups, also present in the ligands, allow the necessary surface charge for stabilization in suspensions. Considering that the size of the QDs is of paramount importance in cellular assays (influencing the cellular internalization of the QDs) and in Förster resonance energy transfer quenching-based methodologies (influencing the distance between the sensor and target), the ligand-exchange approach is the method of choice, comparing with silanization and amphiphilic attachment of ligands, since it allows better control of the diameter of QDs. Nevertheless, some disadvantages include reduced stability of the QDs over time due to formation of disulfides between thiol molecules that detach from the surface, causing oxidation phenomena at the surface and aggregation, and reduction of the quantum yield. These disadvantages were surpassed by using the thiol dihydrolipoic acid (DHLA) or pegylated DHLA.^71^ Yet, stability over time and aggregation problems persist after the purification processes and further functionalization, leading to the use of other alternative and more complex exchange molecules compromising the advantage of simplicity of the process and making it impossible to obtain QDs of smaller size.

Another ligand-exchange approach is using dendrimers, Dendrimer technology can be considered a type of ligand-exchange technique since it is accomplished by the replacement of the hydrophobic ligand present at the QDs’ surface by the dendrimer. Dendrimers are synthetic macromolecules with a globular structure and usually hyperbranched. Polyamidoamine (PAMAM) is a dendrimer that has multiple terminal amino groups and can be used to produce QDs stable in aqueous solutions. The bond to the surface of the QDs is made through the amino groups present in a large number at the PAMAM structure. The amino groups that remain free allow further bioconjugation processes.^72^

Akin et al.^73^ prepared CdSe/ZnS QDs coated by hexadecylamine (HDA) and used PAMAM for the water dispersibility, for cancer cell targeting. The previously synthesized QDs were mixed with the dendrimer and incubated for 15 h, and after the addition of ethyl acetate to the mixture, the complex PAMAM-QDs precipitated. During the reaction, the replacement of the amino groups of the HDA by the PAMAM amino groups occurred. With the use of PAMAM as a surface modifier, it is possible to have several functional groups available for bioconjugation, allowing at the same time the QDs’ electrostatic stabilization. Furthermore, PAMAM due to its chemical constitution, confers high biocompatibility to the nanomaterials, and has a strong buffering capacity, allowing the maintenance of QDs’ fluorescence even under acidic conditions in vesicles.

#### Silanization

Silanization is the encapsulation of the QDs in silica. Besides the excellent properties of silica (chemically inert, nontoxic, and optically transparent), the silica coating allows the protection of QDs from external factors, namely, from oxidation and other chemical processes.^67,74^

Shandilya et al.^75^ synthesized quantum dot-antibody nanoconjugates for the detection of circulating miRNAs in plasma samples. The authors produced CdSe/CdS/CdZnS/ZnS core/multishell QDs, using 1-octadecene (ODE) and ODA (amphiphilic ligands) as caping agents. The core–shell QDs were coated with silica via a modified reverse microemulsion method, to make them water-soluble. The dispersed QDs in toluene were mixed with hexane and the surfactant Brij L4 to form a microemulsion. Then, tetraethyl orthosilicate (silane agent) was added to the mixture, with the aim of replacing the amphiphilic ligands (1-octadecene and ODA) on the surface of the quantum dots. The solution was stirred for 24 h for QDs being coated by the silica shell. To functionalize the silica-coated QDs, with carboxyl groups, carboxyethylsilanetriol (CEST) was added next.^76^ The obtained quantum dots presented an average diameter of 20 nm and a spherical shape, emitting at 617 nm.

Compared with other surface modification methods, this one has the disadvantage of increasing particle size, since silanization can engulf multiple nanoparticles during the shell formation. This phenomenon is dependent on the concentration of particles in suspension, in such a way that more concentrated suspensions originate silica-coated particles of much higher dimensions.^77^ Additionally, in silanization protocol further modification of the shell is needed, as exemplified above in the work of Shandilya et al., in which they added carboxyl groups through CEST to the silica shell. Also, amine groups could be added through the use of (3-(2-aminoethylamino)propyl)trimethoxysilane that contains terminal amine groups.^78^ The modification of the silica shell as described above is necessary to confer QDs with functional chemical groups for further reaction in bioassays and chemical assays.

#### Encapsulation by Amphiphilic Molecules

When quantum dots are synthesized by a solvothermal method, they are capped by hydrophobic ligands, such as TOP and TOPO, that prevent further bioconjugation processes since in this case the QDs are not water compatible. To circumvent this problem, encapsulation of the QDs with amphiphilic molecules can be used instead of exchanging the surface ligands.^79^ In this protocol, the hydrophobic section of the amphiphilic molecules has affinity for the hydrophobic ligands, such as TOP and TOPO, while the hydrophilic side guarantees the water compatibility of the nanomaterials. Overall, there are three main groups of amphiphilic molecules that can be used in the process:^42^ (i) poly(acrylic acid)-based copolymer modified with aliphatic amines such as octylamine, isopropylamine, or dodecylamine; (ii) poly(maleic anhydride) copolymers, such as poly(maleic anhydride-*alt*-1-tetradecene), poly(maleic anhydride-*alt*-1-octadecene), and poly(maleic anhydride-*alt*-1-decene), formed from maleic anhydride and alkenes; (iii) block copolymers formed by linear and sequential arrangements of different polymers.^80^

An example of the application of the surface modification by encapsulation with amphiphilic molecules, poly(maleic anhydride-*alt*-1-octadecene-*co*-poly(ethylene glycol) (PMAO–PEG), is given by Zhao et al.^81^ The authors synthesized PbS QDs capped by three types of capping ligands, oleylamine (OLA), OA, and OA/TOP, and studied the effects on the QDs’ optical properties upon the encapsulation with PMAO–PEG. The PbS QDs were added to the mixture of amphiphilic molecule in chloroform and stirred for 6 h. The authors pointed out that the QDs’ optical properties depended on the original capping ligands and were not influenced by the encapsulation with amphiphilic polymers. The procedures for encapsulation by amphiphilic molecules, for example, PMAO–PEG, generally result in the formation of a novel outside polymer shell increasing thus the hydrodynamic diameter of the final QDs, this being a possible limitation for the use of the nanomaterials in some bioapplications.

#### Cavity–Chain

A molecule with a structure capable of forming a lacuna, in which a ligand present on the QDs’ surface can be linked inside that structure, is called a cavity–chain molecule. These cavity–chain molecules are low molecular weight polymers, such as, for example, cyclodextrins (CD). Through covalent bonding, the cavity–chain polymer wraps the QDs through their surface ligands. Since the cavity structure is hydrophobic and the periphery is hydrophilic, further bioconjugation for applications in the biomedical field are achievable.^66,82^

**Figure 3:**
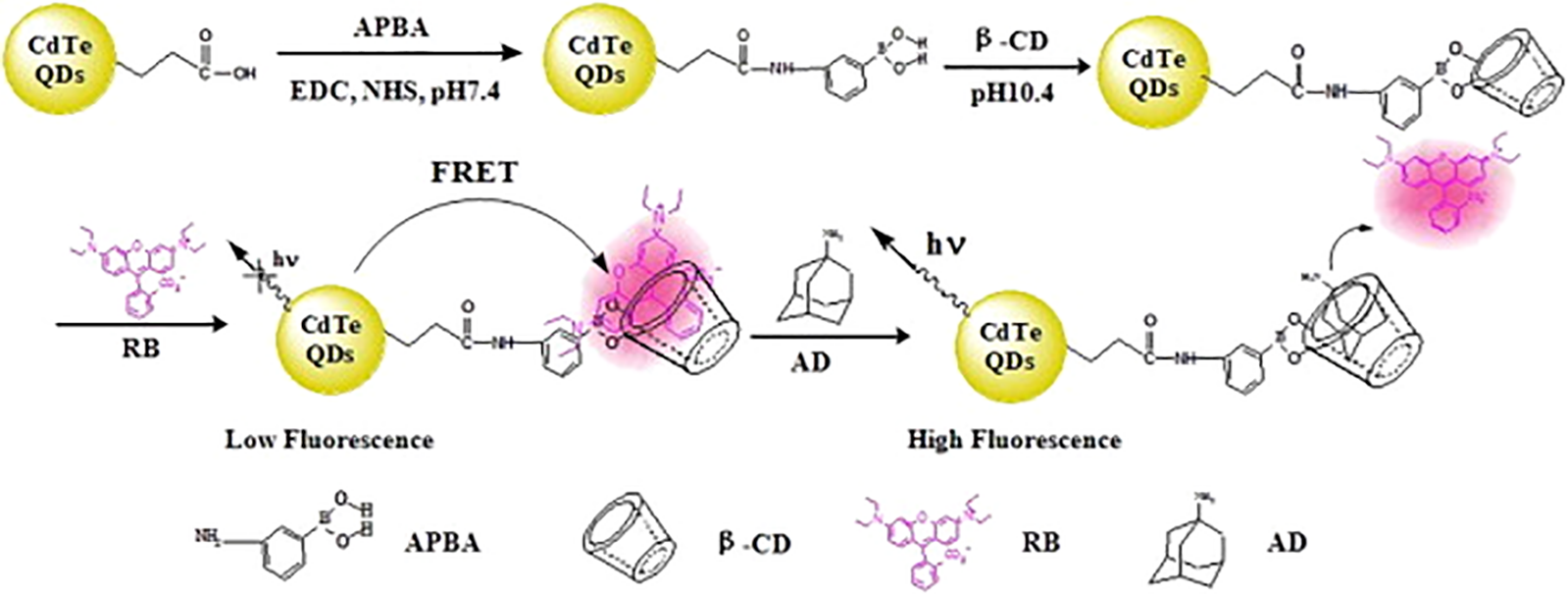
Scheme of the cavity–chain surface modification of QDs. Reprinted with permission from ref (84). Copyright 2012 Elsevier.

β-CDs are cyclic oligosaccharides consisting of seven glucopyranose units, linked by α-1,4 glycosidic bonds, resulting in a 3D cone shape, with an inner open cavity. These are exploited in the development of β-CDs-based nanocarriers with QDs^83^ and bioimaging.^84^ In the work of Ai et al., MPA-CdTe QDs were first prepared and, afterward, reacted with (3-aminophenyl)boronic acid (APBA) by exploiting the EDC/NHS activation chemistry. The APBA molecules served as linkers to β-CDs (Figure 3). The β-CDs-functionalized CdTe QDs, loaded with the anticancer drug amantadine, were tested for internalization in cancer cells HepG2. Through fluorescence imaging it was observed that the developed nanocarrier permeated the cell membrane and, additionally, the drug delivery and release in the cytoplasm.

### Functionalization Approaches

The concepts of QDs’ surface modification and its functionalization are distinct, although they can often be confused. To sum up, surface modifications are the designation given to QDs’ surface alterations necessary to make them appropriate for bioconjugation, also known as the strategies for the hydrophilization of hydrophobic QDs, whereas functionalization is the process of attachment of functional molecules to the QDs, such as, for example, nucleic acids, antibodies, and proteins, among others.^69^

Thus, regarding to the QDs’ functionalization, the linkage between biomolecules and the QDs surface is made by two different types of interactions: covalent and noncovalent binding. The covalent interactions occur when activated functional groups are immobilized on the QDs’ surface through different bioconjugation chemistry, and the noncovalent linkage occurs via hydrophobic, electrostatic, or affinity interactions between some biomolecules and the surface of the QDs.^67,86^ The number of biomolecules that can be immobilized on the surface of the QDs depends not only on the steric hindrance and the size of the QDs but also on the adopted bioconjugation strategy.^67^

In 1998, initial works were encountered involving the biofunctionalization of QDs for bioimaging,^87,88^ enabling the expansion of QDs applications in the biomedical and bioanalysis areas, through the use of surface modifiers such as proteins,^89^ small molecules,^90^ peptides,^91^ or nucleic acids.^92^ The use of nucleic acids sequences, such as deoxyribonucleic acid, to modify the surface of QDs has gained a lot of attention in recent years attributed to some advantages such as good stability, small size, and easily tailor-made to adapt to the analyte targets with great precision and specificity (due to base-pairing), and all of this at low cost nowadays.^93^ These DNA-functionalized QDs have proven to be excellent biosensors and tools of high potential for bioimaging and drug delivery. If aptamers are used rather than DNA, then the range of applications using aptamer-functionalized QDs^93^ is extended to the detection of ions,^94^ proteins,^95^ and small molecules,^96^ besides cancer cell detection,^10^ miRNA real-time imaging,^97^ and drug or small interference RNA (siRNA) delivery.^98^

The assembly of DNA-functionalized QDs can be based on noncovalent bonds, namely, based on high affinity or electrostatic interactions, or covalent bonds.^99^ Some examples of other noncovalent bindings are the biotin–avidin or −streptavidin interaction and immunoglobulin–ligand interaction. Stanisavljevic et al.^100^ synthesized MPA-capped CdTe QDs and afterward were functionalized with streptavidin. Like avidin, streptavidin is a tetrameric protein but with a higher affinity for biotin, an essential vitamin that works like a coenzyme. The complex biotin–streptavidin is widely used for detection strategies.^101^ For the functionalization of the QDs, streptavidin was added to the QDs’ suspension, stirred for 60 min, and then centrifuged. The modified QDs were collected and dissolved in water. The aim of the authors was to study the capacity of the interaction of the synthesized streptavidin-QDs with the biotinylated analytes, such as biotin-modified oligonucleotides: the biotinylated oligonucleotide cancer sequence of BCL-2 and of hepatitis B virus. Another example of application of biotin–streptavidin chemistry to functionalize QDs was reported by Ji et al.,^102^ to detect and quantify miRNA-141. In this work, PEG-stabilized sulfur quantum dots were immobilized on a glass carbon electrode and functionalized with streptavidin. Following, biotin was reacted with a complementary nucleic acid sequence of miRNA-141. By exploiting a DNA-walker-based approach, the authors were able to detect miRNA-141 with a LOD of 1.39 × 10^–15^ mol L^–1^.

So, regarding noncovalent-based functionalization, in the case of high-affinity secondary interactions, such as, for example, the biotin–streptavidin approach, the produced nanomaterials increase their size significantly, impairing cellular assays based on Förster resonance energy transfer (FRET) methodologies.^103^ Additionally, since biotin is naturally present on mammalian cells, it constitutes an obstacle to assays in these cells. Also, the electrostatic interactions occur due to the attraction of oppositely charged species and do not require a complex chemical reaction to establish the linkage. They have the disadvantage of originating nonspecific binding and less stable interactions and, hence, are not applicable to cellular assays.^67^

**Figure 4:**
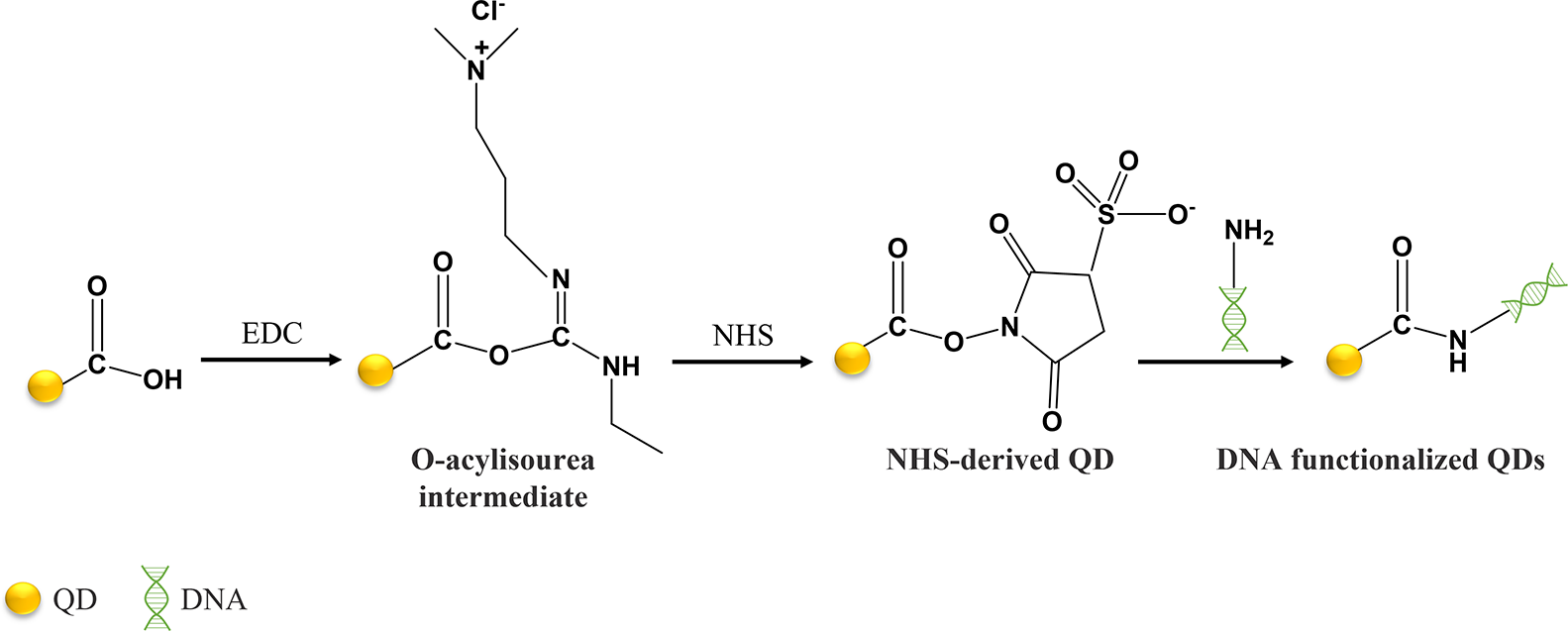
Conjugation of MPA-capped CdTe QDs with amine-modified DNA, via EDC/NHS chemistry.

The covalent bonding is by far the most common conjugation interaction and consists of the reaction between one functional group, namely, sulfhydryl/thiol (-SH) coupling, amino (-NH_2_), or carboxyl (-COOH) groups, present on the surface of QDs, with a ligand, or with another functional group of the biomolecule to be conjugated at the surface. The covalent-based functionalization strategies are thoroughly revised in the scientific literature and are not the aim of the present review.^67,86^ In sum, the covalent coupling can be achieved by exploiting the following: (i) zero-length cross-linkers, such as 1-ethyl-3-[3-(dimethylamino)propyl]carbodiimide hydrochloride (EDC); (ii) homobifunctional cross-linkers, such as dithiobis(succinimidylproprionate); (iii) heterobifunctional cross-linkers, such as *N*-succinimidyl-3-(2-pyridyldithio)propionate; and (iv) trifunctional cross-linkers, such as tris(hydroxymethyl)phosphine. An example of the use of carbodiimide chemistry, using EDC for the functionalization of QDs with DNA, was described by Lv et al.^50^ In fact, EDC is very often selected for the functionalization of carboxyl-modified QDs. The authors synthesized MPA-capped CdTe QDs, and the carboxylic acid functional group was activated with EDC and posteriorly reacted with *N*-hydroxysuccinimide (NHS). The use of NHS allows one to circumvent some disadvantages of using EDC alone, namely, low yield and decrease of QY. Upon the modification of the QDs, an amine-modified DNA strand was mixed with the QDs’ suspension to promote the covalent bonding between the aminated DNA strand and the QDs, via amide conjugation (Figure 4). These DNA-functionalized QDs were used for the detection and quantification of miRNA-122.

## Sensing of Cancer miRNAs Biomarkers

Cancer biomarkers can have different purposes, helping to discriminate a cancer patient from a healthy person, as a very important example. Some of the potential uses for these types of biomarkers are the estimation risk of developing cancer; determination of the prognosis, screening, and monitoring of disease development; the distinction of tumor types (benign and malignant); the evaluation of cancer recurrence, even before patients become symptomatic; the determination of disease progression in the future; and the monitoring of the efficacy of treatment.^104^ miRNAs are a typical example of cancer biomarkers that present distinctive expression profiles (up-regulated or down-regulated) depending on the tumor. For example, miRNA-221 is related with breast cancer, melanoma, and thyroid cancer; miRNA-21 is generally related with breast, colon, and lung cancers; miRNA-155 is associated with lung and breast cancers; miRNA-15 and miRNA-16 are related with B-cell chronic lymphocytic leukemia; and miRNA-223 is associated with acute myeloid leukemia, among others.^105,106^

Therefore, combining the various exclusive physicochemical properties of the QDs with the paramount need for clinical detection of potential cancer biomarkers such as miRNAs, in this section will be presented the state of the art, between 2013 and 2021, about the use of quantum dots for sensing cancer miRNAs biomarkers.

### Electrochemiluminescence Biosensor

Electrochemiluminescence (ECL) is a light-emitting phenomenon, more specifically a type of luminescence that does not require optical excitation.^107^ The ECL mechanism is related with the emission of light by chemical species after undergoing exergonic electron-transfer reactions to generate the electronically excited state of the luminophore. The ECL mechanism can be divided into two dominant pathways: the annihilation and the co-reactant pathways. In the annihilation pathway, the emitter species is oxidized and reduced, producing a radical cation and anion that are annihilated in order to produce excited molecules which emit light. In the co-reactant pathway, the ECL generation is created by an emitter and an assistant reagent, common named as co-reactant. Its function is to be reduced to release an electron and produce its oxidizing intermediate, which will interact with the QDs increasing the ECL signal. The classical known co-reactants are the oxalate ion (C_2_O_4_^2–^), persulfate (S_2_O_8_^2–^), and hydrogen peroxide (H_2_O_2_), among others. Comparing the two pathways, the addition of a co-reactant to drive ECL reactions can enhance the ECL efficiency.^108,109^ In Table 1 were compilated the works found in the literature involving the biosensing of miRNA using QDS, by electrochemiluminescence monitoring.

**Table 1:** Summarized Examples of the Use of Quantum Dots in ECL Biosensors^a^

QDs	λ_max emission_ (nm)	modification method	size (nm)	target	cell line	LOD value (mol L^–1^)	sample	ref
TGA-CdTe		carbodiimide coupling (EDC/NHS)	15.4	miRNA of BRCA1		1.2 × 10^–18^	human serum	(110)
CdS:Mn	∼590		∼5	miRNA-21		11 × 10^–18^	human serum	(111)
				MUC1		0.40 fg/mL		
sulfur	476		3–5	miRNA-21	MCF-7; HeLa	6.67 × 10^–18^		(112)
SnO_2_	650		2–4	miRNA-21	MCF-7; HeLa	2.9 × 10^–18^		(113)
CdTe				miRNA-126		29 × 10^–18^	human serum	(114)
sulfur		biotin-streptavidin	3.3	miRNA-141		1.39 × 10^–15^	human serum	(102)

a TGA, thioglycolic acid; EDC, 1-ethyl-3-[3-(dimethylamino)propyl]carbodiimide hydrochloride; NHS, *N*-hydroxysuccinimide; miRNA, micro ribonucleic acid; MUC1, mucin 1; BRCA1, breast cancer 1 gene mutation.

To trace the amounts of miRNA of breast cancer 1 gene mutation (BRCA1), aiming at an early diagnosis of breast cancer, Yang et al. in 2019^110^ designed an ECL biosensor based on a double signal amplification strategy, providing a novelty in the area of ECL sensors.

Briefly, a DNA hairpin structure, named capture DNA, was immobilized on the surface of a gold electrode, and by the action of double-strand-specific nuclease, the hairpin structure of the capture DNA was cleaved to enable further hybridization. Meanwhile, CdTe QDs were synthesized and functionalized with two sequences of DNA (ssDNA 1 and ssDNA 2), via EDC/NHS chemistry. In the presence of the target miRNA of BRCA1, the DNA strand of the functionalized QDs hybridized with the target, leading to the formation of a 3-QD@DNA NC probe. The 3-QD@DNA NC was later introduced in the modified electrode, hybridizing with the cleaved capture DNA enhancing the ECL signal. However, when target miRNA is absent, no ECL signal is generated.

The sensitivity of the proposed biosensor was assessed and a linear relationship between the ECL signal produced, and the miRNA concentration was obtained ranging from 5 × 10^–18^ to 5 × 10^–15^ mol L^–1^, the LOD value being about 1.2 × 10^–18^ mol L^–1^. To evaluate the capacity of the developed sensor to detect the target in real samples, the authors performed recovery tests with human serum samples, spiked with increased concentrations of the target. As a result, they obtained recoveries ranging from 94.2 to 103%. Considering the obtained results from the evaluation assays, the authors concluded that the proposed device can be applied in clinical diagnosis to detect trace amounts of miRNA of BRCA1 in the earliest stages of human breast cancer.

**Figure 5:**
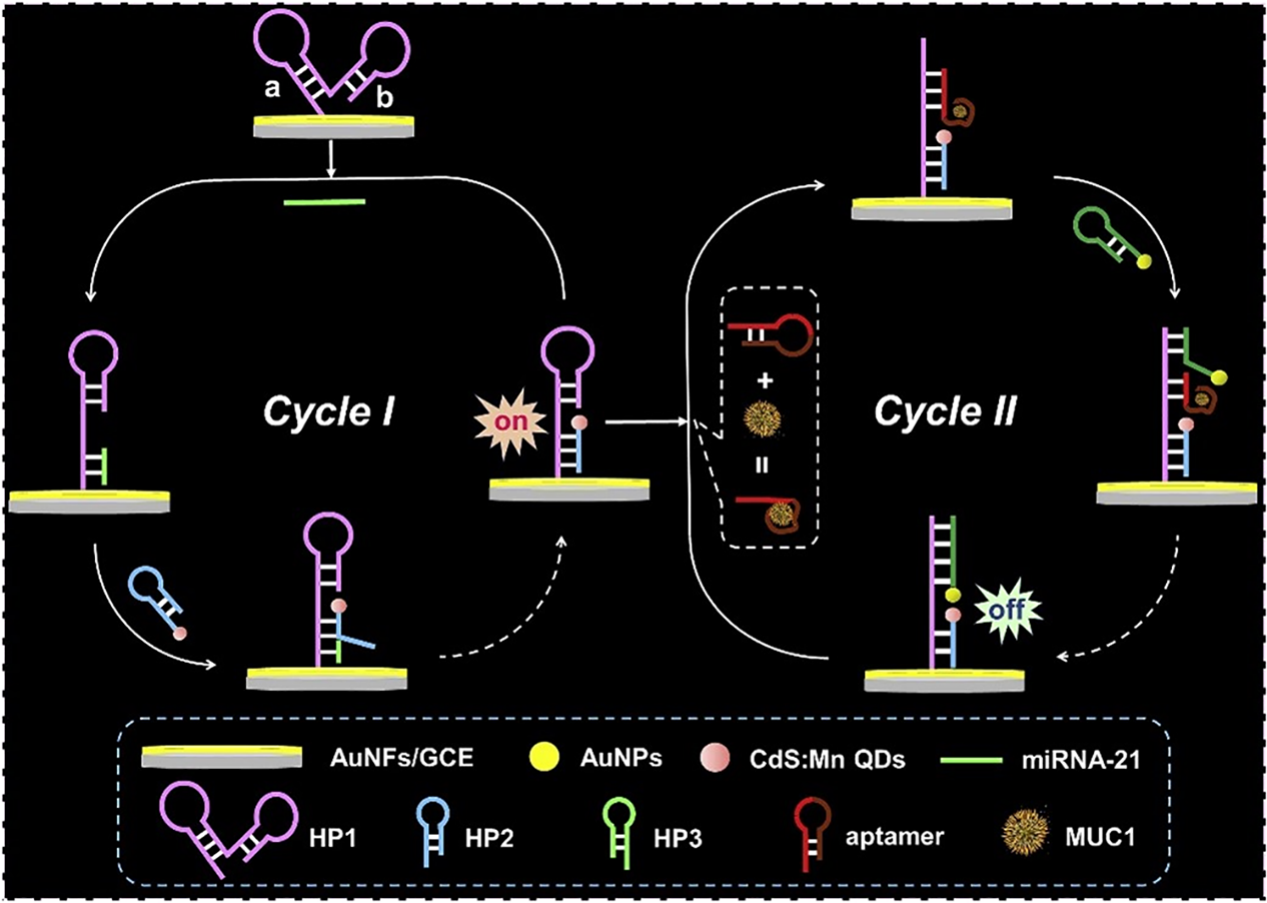
Schematic representation of the principle of the proposed biosensor for miRNA-21 and MUC1 detection, based on DCHA strategy. Reprinted with permission from ref (111). Copyright 2020 Elsevier.

Continuing with the use of electrochemiluminescence signal for cancer-related biomarkers sensing, namely, miRNA-21 and mucin 1 (MUC1), Li et al.^111^ fabricated an ECL plataform based on a dual catalytic hairpin assembly (DCHA). The mechanism of the designed biosensor for the detection of miRNA-21 and MUC1 was composed by two cycles (Figure 5), the first one relative to miRNA-21 sensing and the second one to the MUC1 detection.

For a better understanding of the following process, a DNA hairpin structure probe is a sequence of nucleotides, in which the hybridization between the probe and the target causes the opening of the hairpin structure in the probe, allowing the displacement of the target miRNA-21 by an auxiliary DNA sequence, usually a second DNA hairpin structure probe, completing the amplification procedure by a strand displacement process.

In cycle I, the DNA hairpin probe 1 (HP1), fixed in a GCE decorated with Au nanoflowers, was opened, exposing a DNA segment hybridizing with the target miRNA-21. Then, the hairpin probe 2 (HP2), previously modified by CdS:Mn QDs, was added and hybridized with miRNA-21, forming a tripartite complex of HP1-miRNA-21-CdS:Mn-HP2. Meanwhile, via strand displacement, miRNA-21 was replaced, transforming the previous complex into an HP1-CdS:Mn-HP2. Due to the release of miRNA-21, the process can be repeated, placing a large number of CdS:Mn QDs on the GCE surface conducted to an increase in ECL signal. Hence, an “on” state was reached and cycle I finished, attaining the sensing of miRNA-21. Cycle II was initiated by the binding of MUC1 to the aptamer, allowing the opening of the hairpin in the HP1eCdS:MnHP2 structure, exposing the DNA fragment. Next, AuNPs-modified hairpin probe 3 (HP3) hybridized with the exposed DNA, replacing the MUC1-aptamer binding, to be used in further reaction cycles. This replacement allowed that many AuNPs were placed on the GCE surface. Due to the small distance between the AuNPs and the CdS:Mn QDs, the ECL resonance energy-transfer effect occurred, and the ECL signal decreased, leading to the “off” state. In the meantime, the determination of MUC1 was achieved and cycle II finished.

The performance of the proposed ECL platform was evaluated through different samples spiked with increasing concentration of the target miRNA-21, and the obtained results demonstrated that the higher the concentration of miRNA-21 in the sample, the greater the ECL signal, the LOD being 11 × 10^–18^ mol L^–1^. To determine MUC1 in human serum, miRNA-21 must be measured first, and its concentration must be maintained at 50 × 10^–12^ mol L^–1^. Since the fabricated detection device was composed of two different cycles, where the first one was related to the miRNA-21 detection and the second one was related to the MUC1 detection; the second cycle would only work if the first one was completed. Regarding MUC1 determination, an increase in the concentration of MUC1 originated a decrease in the intensity of the ECL signal, and the detection limit obtained was 0.40 fg mL^–1^.

To evaluate the practicality of the proposed sensor, human serum samples spiked with increased concentrations of miRNA-21 were used, after the exosome lysis pretreatment, and good recoveries were obtained, ranging from 97.90 to 102.14% and from 100.66 to 103.47%, for MUC1 and miRNA-21, respectively. Thus, the authors declared that the developed sensing platform could be used as a tool in clinical diagnosis for the detection of cancer biomarkers, namely, miRNA-21 and MUC1.

Due to the ECL performance of the sulfur QDs (SQDs), and the less toxicity that these ones presented when compared with other heavy metal-based QDs, namely, CdTe, PbS, and CdS, Liu et al.^112^ proposed an ECL biosensor, using SQDs as emitters, and coupled to a DNA walker machine based on triple-stranded DNA (tsDNA) as a signal amplifier, for the detection of miRNA-21.

For the construction of the ECL biosensor, there were three main steps to follow. First is the synthesis of SQDs, followed by an amplification process by duplex-specific nuclease (DSN) enzyme. In a parallel procedure, the target miRNA-21 was cleaved by the DSN on the hairpin, releasing a sequence of DNA (designated here by output DNA). miRNA-21 was also released to serve again as a target and continue the cycling amplification process. As a result, high amounts of output DNA were released to be used in step three. This last step is relative to the construction of the ECL biosensor itself. The previous prepared SQDs were dropped into a glassy carbon electrode, followed by the electrodeposition of gold nanoparticles (AuNPs), and due to the presence of co-reagent S_2_O_8_^2–^, an ECL signal was achieved and conducted to the “on” state. Then, the ferrocene-modified tsDNA was immobilized on the GCE, providing the future action of the DNA walker, and HT was added to the electrode to block the nonspecific adsorption sites. At the same time, the Fc-modified tsDNA induced a decrease on the ECL signal, conducted to the “off” state, because of the quenching effect it had on SQDs. Furthermore, for the action of the DNA walker machine, the output DNA, produced in phase two, hybridizes with the tsDNA, and due to the cleavage action of Mg^2+^, high amounts of fragments of Fc-modified DNA were released, returning to the “on” state by recovering the ECL signal.

**Figure 6:**
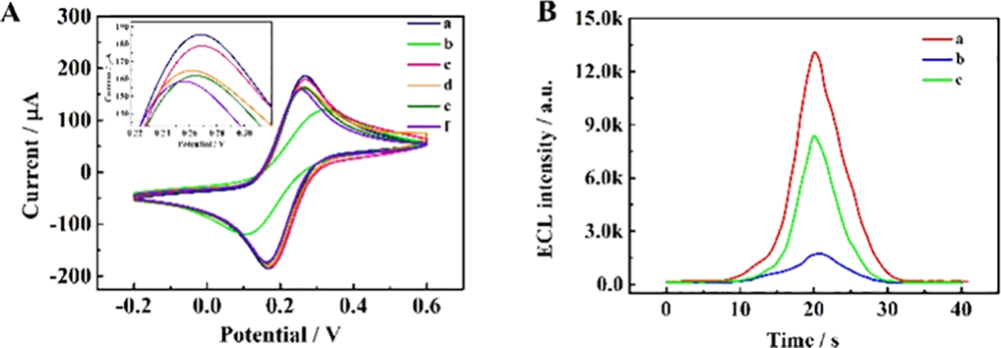
(A) Graphical representation of the CV analysis for the different modified electrodes (a–f): bare GCE (a), GCE/Nafion/SQDs (b), GCE/Nafion/SQDs/AuNPs (c), GCE/Nafion/SQDs/AuNPs/Fc-tsDNA (d), GCE/Nafion/SQDs/AuNPs/Fc-tsDNA/HT (e), and GCE/Nafion/SQDs/AuNPs/Fc-tsDNA/HT/output DNA (f). The inset represents the enlarged partial CV curves. (B) Graphical representation of the ECL signals of the proposed biosensor: GCE/Nafion/SQDs/AuNPs (a), GCE/Nafion/SQDs/AuNPs/Fc-tsDNA/HT (b), and GCE/Nafion/SQDs/AuNPs/Fc-tsDNA/HT/output DNA (c). Reprinted with permission from ref (112). Copyright 2020 American Chemical Society.

The ECL performance of the proposed biosensor was evaluated, and the results obtained are described in Figure 6. In Figure 6A, curve a represents the redox peak of the bare GCE, to be used as comparison with the modified electrodes. The modification of the electrode with SQDs/Nafion (curve b) created a decrease in the current potential, while when AuNPs were electrodeposited on the GCE surface, the intensity on current grew (curve c). However, when the electrode was incubated with Fc-tsDNA, the signal decreased (curves d and e) by the inhibition of the transmission of electrons, and when Mg^2+^ cleaved the Fc-modified DNA, the current decreased (curve f) as expected. Also, the ECL measurements confirmed the previous results (Figure 6B), demonstrating the capacity of the designed biosensor for miRNA-21 sensing. The ECL signal of the AuNPs/SQDs/Nafion-modified electrode is represented in curve a and corresponds to the highest ECL intensity (“on” state). Curve b corresponds to the “off” state, in other words, when the Fc-modified tsDNA were incubated with the electrode and caused the quenching effect. Upon the release of the Fc, the ECL signal increased again (curve c), and the “on” state was achieved.

As conclusion the authors pointed out that the greater the ECL signal, the higher the concentrations of miRNA-21 in the sample, reflecting a linear relationship between the ECL signal and the miRNA-21 concentrations ranging from 20 × 10^–18^ to 1 × 10^–9^ mol L^–1^, with a LOD of 6.67 × 10^–18^ mol L^–1^. The applicability of the sensor in real samples was tested using lysates of MCF-7 and HeLa cells. As a result, and as expected, the expression levels of miRNA-21 were higher in MCF-7 cells, resulting in a high ECL signal, while in HeLa cells, the ECL signal was lower, indicating that, in this type of cancer cells, miRNA-21 was poorly expressed. The obtained results are in accordance with the ones published in the literature, so the authors concluded that the designed sensor can be applied in cancer-related miRNA-21 detection.

Very recently, Yang et al.^113^ developed a ternary electrochemiluminescence (ECL) sensor, for mi-RNA detection, based on SnO_2_ QDs coupled to a 3D DNA walker machine. The 3D DNA walker machine is a type of DNA strategy, constructed at the surface of the quantum dots, or other kinds of nanoparticles, that can be applied as a signal amplification strategy. It is based on the predictability and specificity of Watson–Crick base-pairing and can be from one dimension, two dimensions, or three-dimensions. The last ones, the 3D DNA walker machines, have the capacity to attach more DNA molecules than the one and two dimensions, thereby showing higher walking efficiency and improved signal amplification capability.^115^

**Figure 7:**
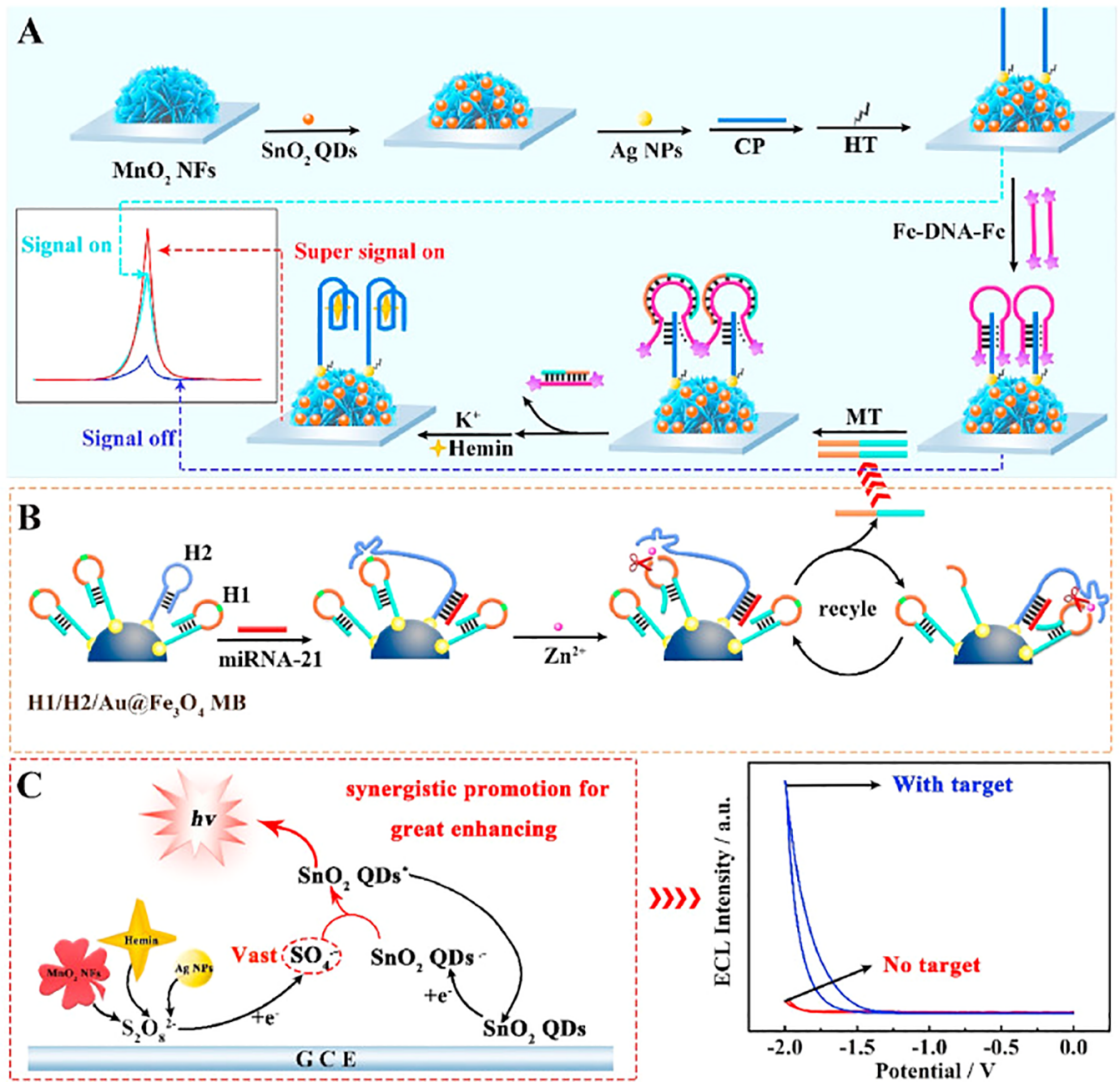
Representation of the designed ECL biosensor for the detection of miRNA-21: (A) Construction process of the biosensor (AgNPs/SnO_2_ QDs/MnO_2_ NFs); (B) 3D DNA walker amplification procedure; (C) synergistic promotion strategy (CP, capture probes; HT, hexanethiol; MT, mimic targets). Reprinted with permission from ref (113). Copyright 2021 Elsevier.

The SnO_2_ QDs are known to have low luminescent intensity due to the wide band gap between the valence band and the conduction band, so to enhance their ECL signal, facilitating the reaction between luminophores and co-reactants, a combination of co-reaction accelerators, such as MnO_2_ nanoflowers (MnO_2_ NFs), silver nanoparticles (AgNPs), and hemin/G-quadruplex, were selected. The proposed “on–off–super on” biosensor was composed of three nanocomposites (AgNPs/SnO_2_ QDs/MnO_2_ NFs) connected through electrostatic interactions. The schematic representation of the assembly is presented on Figure 7.

The AgNPs and the MnO_2_ NFs acted like an accelerator of the reaction between luminophore (SnO_2_ QDs) and co-reactant (S_2_O_8_^2–^). By accelerating the efficiency of this reaction, the ECL signal from the luminophore increases, because they promote more easily the reduction of S_2_O_8_^2–^, by generating more intermediates. These intermediates will, in turn, react with SnO_2_ QDs, amplifying the ECL signal (Figure 7C).

In the first phase, the “on” state was achieved after the assembly of the biosensor, accelerator–luminophore–accelerator (Figure 7A) at the surface of the bare glassy carbon electrode (GCE), conducting to the first ECL signal. Then, in the second phase, when the double-labeled ferrocene quencher probes (Fc-DNA-Fc), which are electrochemically active probes, were added, they hybridized with the capture probes and some triplex DNA structures were formed, leading to the quenching effect, achieving, in this way, the “off” state. Afterward, a parallel procedure was conducted, based on the 3D DNA walker amplification (Figure 7B), in which miRNA-21 was converted to single-stranded fragments, named as mimic targets, to continue the third phase of the sensing process. After the incubation of the mimic targets, the Fc-DNA-Fc was removed from the surface of the GCE and the hemin was added, leading to the formation of the third co-reaction accelerator, the hemin/G-quadruplex complex. These structures reached a super high ECL signal corresponding to the “super on” state.

**Figure 8:**
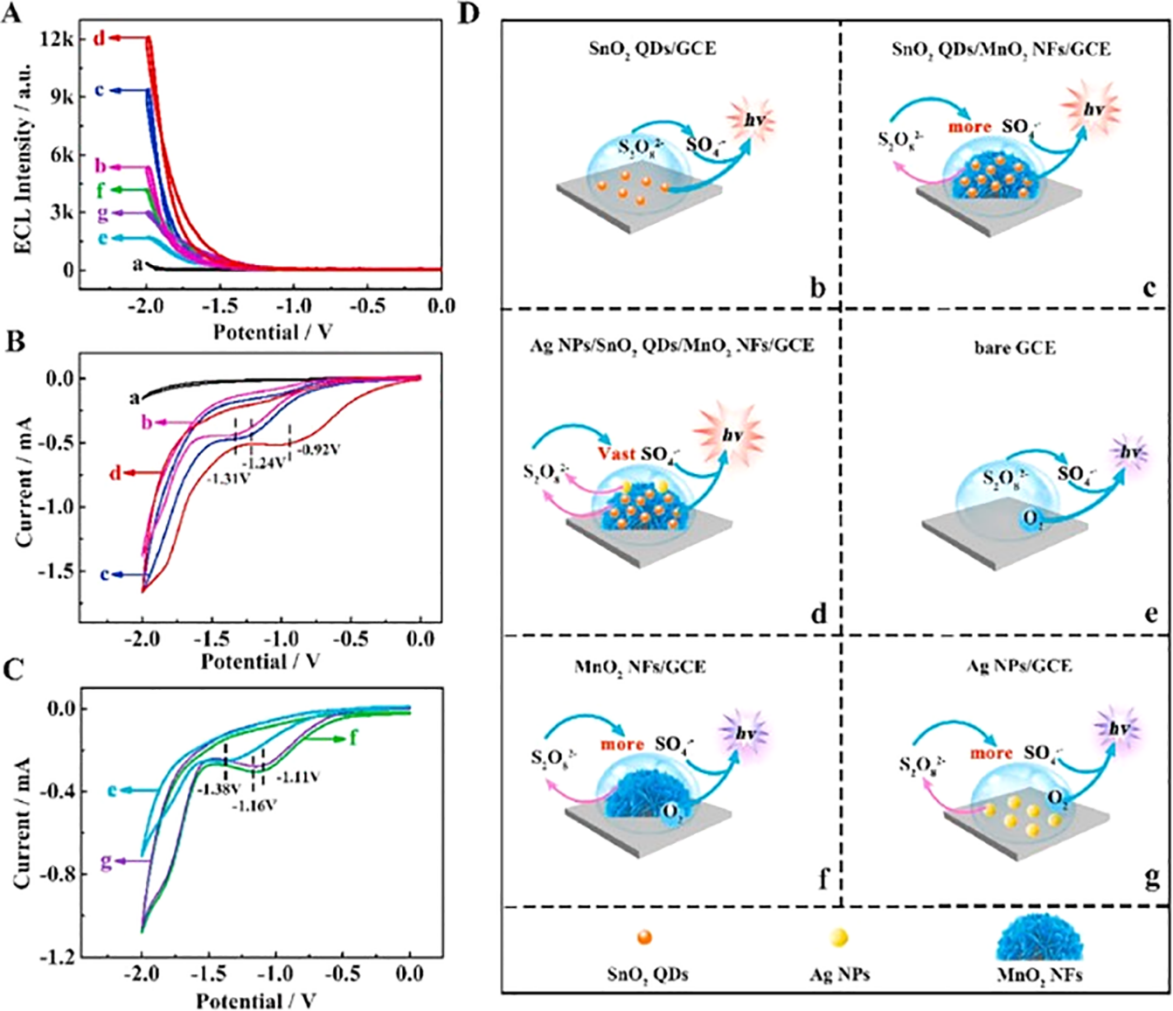
Schematic representation of the ECL biosensor. (A) Graphic representation of the ECL-potential profiles. (B, C) Cyclic voltammetry of the modified electrodes: SnO_2_ QDs/GCE in PBS solution (a), SnO_2_ QDs/GCE (b), SnO_2_ QDs/MnO_2_ NFs/GCE (c), AgNPs/SnO_2_ QDs/MnO_2_ NFs/GCE (d), bare GCE (e), MnO_2_ NFs/GCE (f), and AgNPs/GCE (g) in S_2_O_8_ in 0.1 M PBS (pH 7.4). (D) Representation of the mechanism of luminescence of the different modified electrodes. Reprinted with permission from ref (113). Copyright 2021 Elsevier.

To illustrate the ECL enhancement mechanism of the proposed biosensor, the authors performed an assay in which the ECL intensity and the current generated by the sensor were measured. The measurement of the produced current was to see how much potential had to be applied for the co-reactant species to be reduced. Different modified electrodes were used, and the obtained results are in Figure 8. The weakest curve (curve a, Figure 8A), representative of SnO_2_ QDs/GCE in PBS solution, only presented an intensity of 325 au. This intensity was produced by SnO_2_ QDs when they were in the excited state. When the co-reactant S_2_O_8_^2–^ was added (curve b, Figure 8A) an increase in the intensity to 5356 au was observed. At the same time, comparing the measurement of cyclic voltammetry (CV) (Figure 8B), the reduction of S_2_O_8_^2–^ led to a significant reduction on the current signal, showing a peak at −1.31 V (curve b, Figure 8B). Regarding to the ECL signal of SnO_2_ QDs/MnO_2_ NFs/GCE (curve c, Figure 8A), it is possible to verify that there was an increase to 9698 au when compared with the previous one, and the current became more positive, registering a potential of −1.24 V (curve c, Figure 8B). With the addition of AgNPs, and completing the assembly of the biosensor, the ECL intensity was the strongest registered (curve d, Figure 8A), with 12106 au. Like the previous electrode (SnO_2_ QDs/MnO_2_ NFs/GCE), also with this one (AgNPs/SnO_2_ QDs/MnO_2_ NFs/GCE), the current potential became even more positive, −0.92 V (curve d, Figure 8B). This means that the combination of the co-reaction accelerators promotes a synergistic effect, and it is not necessary to apply a strong working potential to reduce S_2_O_8_^2–^ and obtain a high ECL signal.

Curves e, f, and g in Figure 8A are related to the analysis of the interactions between MnO_2_ NFs and AgNPs with S_2_O_8_^2–^. Comparing the intensity of bare GCE in S_2_O_8_^2–^ solution (curve e in Figure 8A) with the MnO_2_ NFs/GCE also in S_2_O_8_^2–^ solution (curve f, Figure 8A), there was a 2.4-fold increase from the first measurement to the last. Furthermore, in the CV curves, there was a positive shift from −1.38 V (curve e, Figure 8C) to −1.16 V (curve f, Figure 8C), and the peak current increased. The same analysis was performed with the electrode upon the addition of AgNPs, and similar results were obtained. Thus, the authors showed that the addition of these two co-reaction accelerators promoted a synergistic effect and improved the ECL intensity of the SnO_2_ QDs, due to the interaction of them with S_2_O_8_^2–^, facilitating the S_2_O_8_^2–^ reduction producing a large amount of the intermediates (SO_4_^•–^). As explanation, for all of the improvements achieved by the combination of co-reaction accelerators, the authors pointed out that MnO_2_ nanoflowers (the first co-reaction accelerator), besides acting like a co-reactant and reducing S_2_O_8_^2–^, provided a matrix capable of capturing various SnO_2_ QDs, leading to the enhancement of ECL signal, and the AgNPs (the second co-reaction accelerator) allowed the creation of several catalytic active sites where S_2_O_8_^2–^ could be reduced.

When the biosensor performance to detect the miRNA-21 was evaluated, a LOD of about 2.9 × 10^–18^ mol L^–1^ was obtained. With this low value, the authors confirmed the highly sensitive miRNA detection capacity from the designed approach, compared with other published works. To investigate the applicability for clinical diagnosis in cancer cells, the authors tested the biosensor in real samples, selecting MCF-7 and HeLa cells to accomplish the study. As expected, the ECL sensor reported a high ECL response, referent of a higher expression of miRNA-21 in MCF-7 cells, than in HeLa cells.

### Photoelectrochemical Biosensor

In the photoelectrochemical (PEC) process, the light, originating from an excitation external source and focusing on a photoelectrode or other material, triggers a photocurrent signal. In a PEC biosensor, photoactive materials are deposited in an electrode surface and, when they are irradiated with light, they generate an electric current, which is used as a signal readout.^116^

The use of PEC-based biosensors as miRNA detection tools has become common in the recent years, due to their features, namely, low background signal and good sensitivity, beyond their simplicity and possibility of miniaturization.^117,118^ In Table 2 the works found in the literature involving the biosensing of miRNA using QDs by photoelectrochemical monitoring were compiled.

**Table 2:** Summarized Examples of the Use of Quantum Dots in PEC Biosensors^a^

QDs	λ_max emission_ (nm)	capping	modifications and functionalization	modification method	size (nm)	target	cell line	LOD value (mol L^–1^)	ref
CdSe		TGA	DNA	carbodiimide coupling (EDC/NHS)	3	miRNA-21		5.6 × 10^–15^	(119)
CdTe					4	miRNA-141		33 × 10^–18^	(120)
CdTe		MPA	C_3_N_4_; 3D graphene hydrogel	carbodiimide coupling (EDC/NHS)		miRNA-21; miRNA-141			(121)
CdTe		MPA	CeO_2_	carbodiimide coupling (EDC/NHS)		miRNA-141		0.17 × 10^–15^	(122)
CdSe		MPA	nitrogen-doped porous carbon-ZnO polyhedral		5	miRNA-155		49 × 10^–18^	(117)
ZnS		MPA	manganese-doped cadmium sulfide		3	miRNA-141		3.30 × 10^–15^	(123)
PbS		TGA	DNA	carbodiimide coupling (EDC/NHS)	5	miRNA-21		0.57 × 10^–15^	(124)
CdTe	526	MPA	DNA	carbodiimide coupling (EDC/NHS)	3.0 ± 0.8	miRNA-21		0.37 × 10^–15^	(125)
CdTe		MPA	DNA		5	miRNA-21	MCF-7; HeLa	3.3 × 10^–15^	(49)
CdS		MPA	DNA	carbodiimide coupling (EDC/NHS)	4	miRNA-21	breast cancer	6.6 × 10^–15^	(126)
						let-7a		15.4 × 10^–15^	
CuS		TGA	DNA	carbodiimide coupling (EDC/NHS)		miRNA-21		3.25 × 10^–15^	(127)
CdS					3	miRNA-141		0.33 × 10^–15^	(128)

a TGA, thioglycolic acid; MPA, 3-mercaptopropionic acid; DNA, deoxyribornucleic acid; EDC, 1-ethyl-3-[3-(dimethylamino)propyl]carbodiimide hydrochloride; NHS, *N*-hydroxysuccinimide; miRNA, micro ribonucleic acid.

A PEC biosensor was developed by Cong et al.^119^ in 2018, for miRNA-21 detection using CdSe QDs as sensitizers. The basis of the PEC biosensor was composed of TiO_2_ nanotubes (TiO_2_ NTs), reduced graphene oxide (RGO), and gold nanoparticles (TiO_2_NTs/RGO/AuNPs electrode). CdSe QDs, functionalized with a hairpin structure of DNA, were immobilized on the TiO_2_NTs/RGO/AuNPs electrode surface. Under light excitation, this conjugation provided the enhancement of the PEC signal since the distance between the CdSe QDs and the electrode surface was close enough to produce the sensitization effect, i.e., effectively completing the electron transference. However, in the presence of the target miRNA-21, the hairpin structure of the DNA opened, hybridizing with miRNA-21. Consequently, the photocurrent intensity decreased because the distance between the CdSe QDs and the electrode surface increased, due to the change in the conformation of the DNA strand, which became straight and longer. These variations in the PEC signal allowed the detection of the target miRNA-21.

The detection performance of the designed approach was evaluated, and the authors obtained a LOD of 3.6 × 10^–15^ mol L^–1^. To test the performance of the developed sensor in real samples, the authors performed an assay using human serum, and they obtained recoveries ranging from 91.8 to 108.2%, for increasing concentrations of miRNA-21. With these results, the authors expected that the developed sensing device could be used as a detection tool in clinical diagnosis to detect miRNA-21.

CdTe quantum dots were used together with poly[4,8-bis[5-(2-ethylhexyl)thiophen-2-yl]benzo[1,2-*b*:4,5-*b*′]dithiophene-2,6-diyl-*alt*-3-fluoro-2-[(2ethylhexyl)carbonyl]thieno[3,4-*b*]thiophene-4,6-diyl) (PTB7-Th) and combined with DSN-assisted target cycling amplification, to create a PEC biosensor for miRNA-141 monitoring, in 2018 by Li et al.^120^ The DSN-assisted target cycling amplification is an enzymatic strategy to transform target miRNA-141 into large amounts of DNA products, previously mentioned as output DNA, which was used later in the assembly process of the PEC biosensor, explained below.

In this biosensor, the PTB7-Th was a donor–acceptor type photoactive material and the CdTe QDs acted as a sensitizer, where the combination of both led to an initial photocurrent response. Briefly, PTB7-Th was added into the glassy carbon electrode, followed by a layer of CdTe QDs and a layer of gold nanoparticles. Then, a sequence of nucleotides with a hairpin structure named as H2 was incubated into the modified electrode. In parallel, through the DSN-assisted target cycling amplification, as mentioned above, output DNA was created and was used to change the conformation of the H2 structure, opening the hairpin and hybridizing. Afterward, some DNA strands, S1 and S2, continued the hybridization, creating a DNA “super sandwich” structure capable of loading huge amounts of MnPP. Through biocatalytic precipitation, in the presence of H_2_O_2_ and 4-CN, a benzo-4-chlorohexadienone (4-CD) precipitate was formed on the electrode surface. Under the excitation of UV light, this 4-CD precipitate has quenching activity, not a complete electron transmission path by steric hindrance, preventing the electrons transfer to O_2_, which resulted in a significant decrease in the PEC signal, reaching a maximum of 91% of quenching. Consequently, miRNA-141 can be detected. The authors observed that the higher the miRNA-141 concentration in the sample, the lower the PEC signal, the calculated LOD being 33 × 10^–18^ mol L^–1^. With this sensing platform, the authors created a different type of PEC biosensor through the formation of a precipitate on the surface of the modified electrode; however, the written work did not contain a described application assay to evaluate the performance in real samples.

For the first time, in 2019, Hao et al.^121^ accomplished the fabrication of a PEC biosensor for the dual detection of miRNA-141 and miRNA-21. The authors recognized the importance of the construction of a sensor that could detect simultaneously two different analytes, allowing the reduction in analysis time, reagents, and sample volume, and cost, per analysis. This biosensor was composed of two types of nanomaterials working as active PEC materials: (i) CdTe loaded carbon nitride nanosheets (CdTe-C_3_N_4_) and (ii) CdTe loaded 3D graphene hydrogel (CdTe-3DGH), which generated two distinct photocurrents, anodic and cathodic, respectively. The changes in the photocurrent signal allowed the determination of the target miRNAs.

The assembly of the biosensor, as well as the detection mechanism of the targets, are described as follows. An indium tin oxide electrode was divided into two parts, in which CdTe-C_3_N_4_ and CdTe-3DGH were deposited separately, creating a PEC signal. Afterward, via carbodiimide chemistry, a single-strand sequence of DNA, named by the authors as probe DNA 1, was linked to CdTe-3DGH, and a second single-strand sequence of DNA, designated as probe DNA 2, was linked to CdTe-C_3_N_4_, leading to a reduction of the initial PEC signal because of the enhanced steric hindrance. Later, when present in the sample, miRNA-141 hybridized with probe DNA 1, while miRNA-21 hybridized with probe DNA 2, but in a competition of hybridization reaction with the AuNPs functionalized with a complementary sequence of DNA. At this moment, the photocurrent increased again due to the surface plasmon resonance of gold nanoparticles. In conclusion, the greater the amount of target miRNA present in the sample, the smaller the PEC signal.

Recovery tests were performed in spiked human serum to test the capacity of the proposed biosensor to be used in real samples. As a result, the authors obtained recovery values ranging from 98.8 to 102.7% for miRNA-141 and from 98.5 to 102.9% for miRNA-21, which made them conclude that the designed approach can serve as an analytical tool in the detection and determination of miRNA (miRNA-141 and miRNA-21) in real samples.

The detection and determination of miRNA-141 were achieved by Li et al.^122^ in 2019, when they used a PEC biosensor based on a CdTe QD-CeO_2_ complex, where CeO_2_ was the photoactive material and the CdTe QDs were the sensitizer. The combination of these two materials generated a photocurrent response, which was decreased in the presence of the target, because miRNA-141 caused, indirectly, a quenching effect.

**Figure 9:**
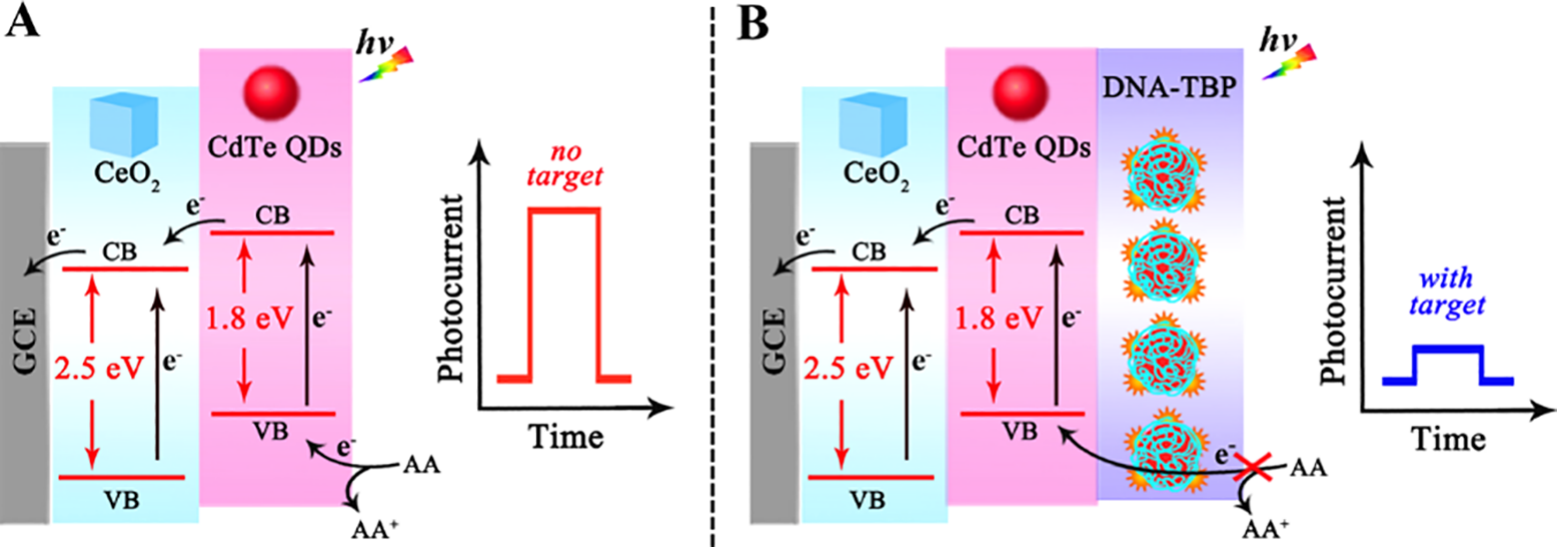
Representative scheme of the mechanism of PEC biosensor signal: (a) in the absence of the target and (b) in the presence of the target. Reprinted with permission from ref (122). Copyright 2019 American Chemical Society.

Briefly, the PEC biosensor was composed of a glassy carbon electrode, where a layer of gold nanoparticles was deposited, and then the CdTe QDs were immobilized. After that, a nucleotide sequence with a hairpin structure, designated as HP1, was added to the surface of the modified electrode. The amplification process was made through a DNA walker system, in which small amounts of miRNA-141 were transformed into various sequences of output DNA. In the presence of this output DNA, the hairpin structure of HP1 was opened and hybridized with it. Then, with the addition of another two sequences of nucleotides, designated as DNA 3 and 4, a super sandwich DNA structure was formed due to the hybridization process. After all, the TATA-binding protein (TBP) was added and complexed with the DNA structure producing a DNA–TBP complex, which led to a substantial reduction of the photocurrent, due to the steric hindrance created by the super sandwich structure, preventing the electron transference between the CdTe QD-CeO_2_ and the electron donor, ascorbic acid. In Figure 9A is represented the signal generated by the PEC biosensor in the absence of the target miRNA-141, and in Figure 9B is the representation of the mechanism of quenching of the PEC signal, when miRNA-141 is present in the samples.

The analytical performance of the proposed platform was evaluated, and the authors verified that the increase in miRNA-141 levels led to a decrease in the PEC signal, obtaining a linear relationship ranging from 0.5 × 10^–15^ to 5 × 10^–9^ mol L^–1^, and the calculated LOD was 0.17 × 10^–15^ mol L^–1^.

In summary, the authors concluded that it was a promising sensing platform to be used in clinical diagnosis for miRNA detection.

Also in 2019, Meng et al.^117^ developed another PEC biosensor, but this time for the detection of miRNA-155. In this work, the novel combination of CdSe QDs and N-doped porous carbon-ZNO polyhedral (NPC-ZNO), both materials with interesting photoelectronic properties, resulted in a PEC-based sensor of improved performance. The assembly of the biosensor was as follows: the indium tin oxide electrode was coated with poly(diallyldimethylammonium chloride) (PDDA)-modified reduced graphene oxide (P-rGO), and then the CdSe QDs were immobilized in the electrode surface. After that, a nucleotide sequence with a hairpin structure, designated as HP1, was added to the modified electrode and, in the presence of the target miRNA-155, the hairpin structure of HP1 was opened and hybridized with the target. Another nucleotide sequence with a hairpin structure, named as HP2, previously labeled with biotin, was added to the electrode, to replace the miRNA-155, and hybridized with the exposed sequence of HP1. Thus, miRNA-155 was released to initiate another cycle, and so on, allowing the amplification of the cycle. Later, a streptavidin (SA)-labeled AuNPs/NPC-ZnO nanocomposite reacted with the biotin of the HP2 and become attached to the electrode surface, switching the direction of the photocurrent. The greater the concentration of miRNA-155 in the sample, the greater the response of the photocurrent.

Upon the evaluation of the analytical performance of the proposed PEC biosensor, the authors obtained a linear relationship between the concentration of miRNA-155 and the signal of the photocurrent in a range of 0.1 × 10^–15^ to 10 × 10^–9^ mol L^–1^, the LOD being calculated as 49 × 10^–18^ mol L^–1^. To test the practicality of the designed sensing scheme, the authors chose human serum samples to perform recovery tests, obtaining recoveries ranging between 93.4 and 102.3%.

In a conclusion, the authors stated that their detection platform could be used as a starting point to further explore PEC photocurrent-direction-switching systems for miRNA detection.

For the detection and determination of miRNA-141, in 2019, Mo et al.^123^ developed an “on–off–on” PEC biosensor where manganese-doped cadmium sulfide coupled with zinc sulfide quantum dots (Mn:CdS@ZnS QDs) were used as photoelectric material and manganese porphyrin (MnPP) was used as photosensitizer. The mechanism of action of this sensor did not involve enzymes for the amplification signal, but a cascaded quadratic amplification strategy. The concomitant use of Mn:CdS@ZnS QDs and MnPP enhanced the photocurrent response of the proposed sensing platform.

**Figure 10:**
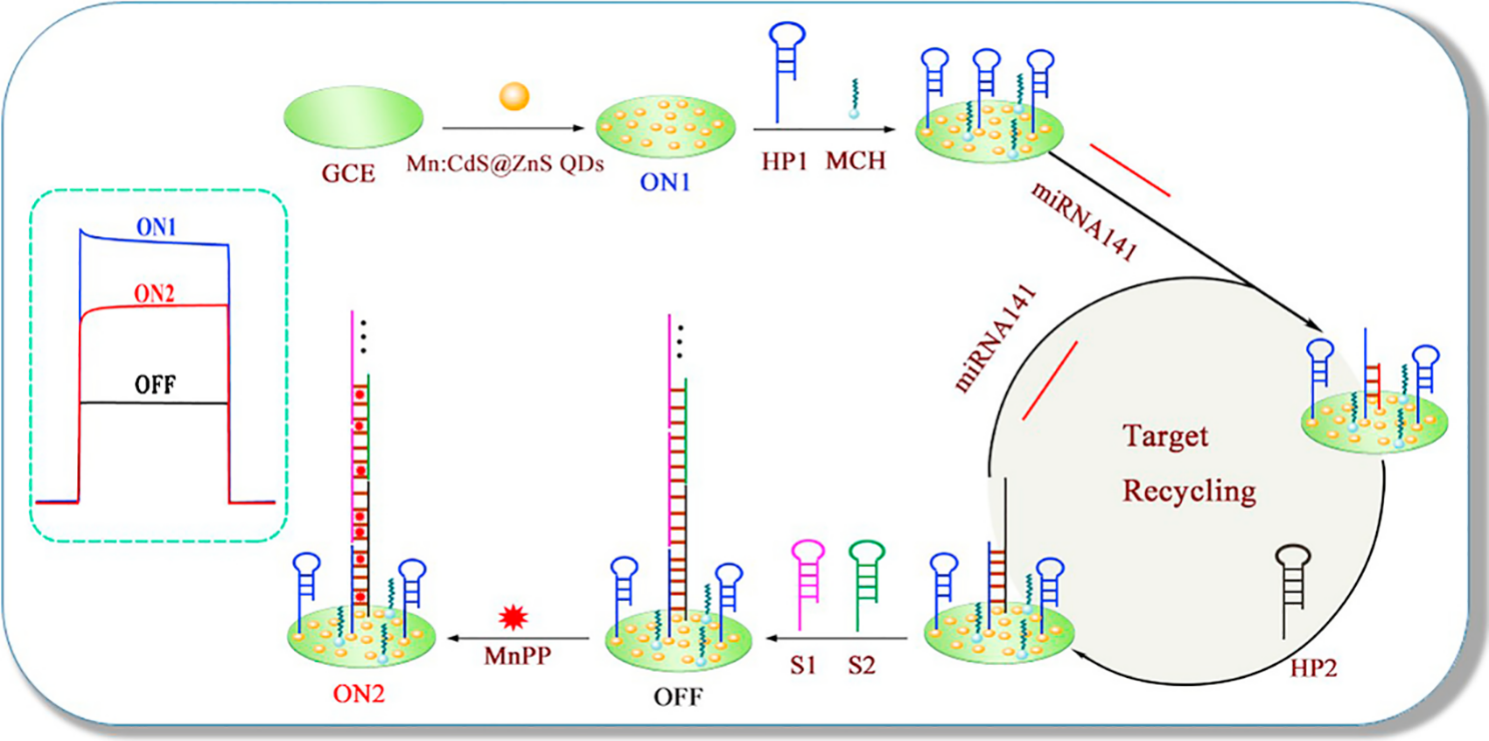
Representative scheme of assembly of the “on–off–on” PEC biosensor. (S1, represents DNA 1; S2, represents DNA 2). Reprinted with permission from ref (123). Copyright 2019 Elsevier.

The schematic representation of the assembly of this biosensor is illustrated in Figure 10. Briefly, the Mn:CdS@ZnS QDs, previously prepared by a hydrothermal synthesis, were immobilized in a glassy carbon electrode producing a high-current signal, denominated “ON1”. Then, the electrode was modified with a hairpin DNA structure, HP1, and when the target miRNA-141 was present, the hairpin structure of HP1 was opened and the miRNA-141 hybridized with it. Then, a second hairpin DNA structure, HP2, with a complementary sequence of HP1, was added to be hybridized with HP1, replacing the miRNA-141 and releasing the target to enable another hybridization event. Afterward, to proceed with the sensing scheme, two more different hairpin structures of DNA, named by the authors as DNA 1 and DNA 2, were incubated with the modified electrode. DNA 1 was complementary of HP2 and hybridized with it, exposing a sequence complementary of DNA 2, which allowed the change in DNA 2 conformation to hybridize with it. In the end of this process, the photocurrent decreased, and the “off” state was reached. Lastly, MnPP was dropped into the electrode, leading to an increase in the PEC signal, recovering the “on” state, denoted “ON2″, due to the photocatalysis and photosensitization of MnPP.

The authors obtained a linear relationship between the photocurrent signals and the miRNA-141 concentrations between 1.00 × 10^–14^ and 1.00 × 10^–8^ mol L^–1^, and a LOD of 3.30 × 10^–15^ mol L^–1^. The designed biosensor was tested in spiked human serum samples allowing recoveries between 95.6 and 106.0%.

In conclusion, in this work the authors presented a novelty in the PEC biosensors for miRNA monitoring, introducing the cascaded quadratic amplification strategy coupled catalytic hairpin assembly with a hybridization chain reaction.

In 2020, Yu et al.^124^ proposed for the first time the construction of a PEC biosensor for miRNA-21 detection, composed of Cu_2_MoS_4_ and In_2_O_3_. This In_2_O_3_@Cu_2_MoS_4_ nanocomposite exhibited a strong initial PEC signal, under visible light, due to the promotion of the separation of electrons and hole pairs, which was decreased later by the introduction of TGA-capped PbS QDs on the electrode surface. The PbS QDs acted like a quencher, competing with the In_2_O_3_@Cu_2_MoS_4_ for light and electrons donors.

Upon visible light excitation, the Cu_2_MoS_4_ electrons from the valence band were excited to the conduction band, leading to the generation of holes in the valence band. Then, the electrons from the conduction band of Cu_2_MoS_4_ were injected in the conduction band of In_2_O_3_, being later transferred to the electrode surface, creating a photocurrent signal (initial high PEC signal). Meanwhile, the holes generated from the valence band of In_2_O_3_ were transferred to the valence band of Cu_2_MoS_4_, and the holes generated in the valence band of Cu_2_MoS_4_ were consumed by the ascorbic acid. When the PbS QDs were present, due to the approximation of the PbS QDs from In_2_O_3_@Cu_2_MoS_4_ nanocomposite, the PbS QDs competed between them for light energy, leading to a decrease in the photocurrent response (low PEC signal – signal off).

The preparation of the “signal-off” biosensor can be described as follows: the surface of In_2_O_3_@Cu_2_MoS_4_ nanocomposite was modified by the addition of TGA, via carbodiimide chemistry (EDC/NHS), to create the necessary carboxyl groups on the electrode surface, which will allow the connection with a sequence of DNA, named hairpin probe 1 (HP1). Later, in the presence of miRNA-21, the hairpin structure of HP1 was opened and hybridized. Afterward, a second DNA hairpin structure (HP2), which includes a fragment of a complementary sequence to HP1, as well as being functionalized with the PbS QDs, hybridized with free HP1 and allowed the release of miRNA-21, through the strand displacement mechanism, causing the insertion of the QDs into the double-stranded DNA. Due to the release of miRNA-21, this process can be repeated, establishing an amplification cycle.

After the analysis of the developed sensor, the authors verified that when the miRNA-21 concentration increased, the photocurrent was reduced, presenting a LOD of about 0.57 × 10^–15^ mol L^–1^. The authors used human serum samples spiked with different concentrations of miRNA-21 and obtained recoveries between 94.0 and 102%, which confirmed the possible application in clinical diagnosis. However, the authors pointed out the electrode modification procedure is difficult to perform, implying that it would have to be improved aiming at clinical diagnosis on a routine basis.

The detection of miRNA-21 was attained by Fu et al.,^125^ in 2020, upon the fabrication of a photoelectrochemical sensor composed of indium tin oxide/TiO_2_/AuNPs photoanode functionalized with CdTe QDs. The authors tested different methods for the construction of the biosensor and concluded that the best procedure was by the deposition of TiO_2_ by spin-coating tetraisopropyl titanate on ITO slices, followed by in situ electrodeposition of AuNPs, and finishing with thermal annealing treatment. The electrode surface was modified with NH_2_-DNA, a sequence of DNA with a hairpin structure modified with NH_2_, to enable functionalization with the CdTe-COOH QDs, via EDC/NHS chemistry. In the absence of the target miRNA-21, and even in the presence of an external light source, the PEC signal was low; however, when miRNA-21 was present, also with the incidence of light, an increase in the photocurrent intensity occurred due to the increasing of the distance between the CdTe QDs and the AuNPs, since miRNA-21 opened the hairpin structure of the DNA probe and hybridized.

Like in the above-mentioned PEC biosensors, the intensity of the signal detected was related to the concentration of the miRNA-21 in the samples. The authors obtained a linear relationship between the photocurrent generated and the miRNA-21 expression levels in the range of 1 × 10^–15^ to 1 × 10^–9^ mol L^–1^, and a LOD value of 0.37 × 10^–15^ mol L^–1^. To test the applicability in real samples, the methodology was subject to an assay with human serum samples. As a result, the PEC biosensor displayed recoveries ranging from 96.3 to 106%, reaching the detection of the target analyte.

Hence, the authors concluded that the proposed device had the potential to be used as a tool in biomedical research for the detection of trace amounts of miRNA-21 and helping in the early diagnosis of cancer.

Another PEC biosensor was developed for miRNA-21 detection by Yuan et al.^49^ in 2020. This bioassay was based on CdTe QDs sensitizing Bi_2_Te_3_ nanosheets combined with DNA amplifying approaches. The novelty in this work, when compared with other PEC biosensors, was the use of Bi_2_Te_3_ nanosheets as part of the biosensor. The Bi_2_Te_3_ nanosheets have excellent electronic properties that make researchers interested in their use, namely, high specific surface areas, and insulator properties due to conductive surface states that allow a restrained electric conduction at room temperature.

**Figure 11:**
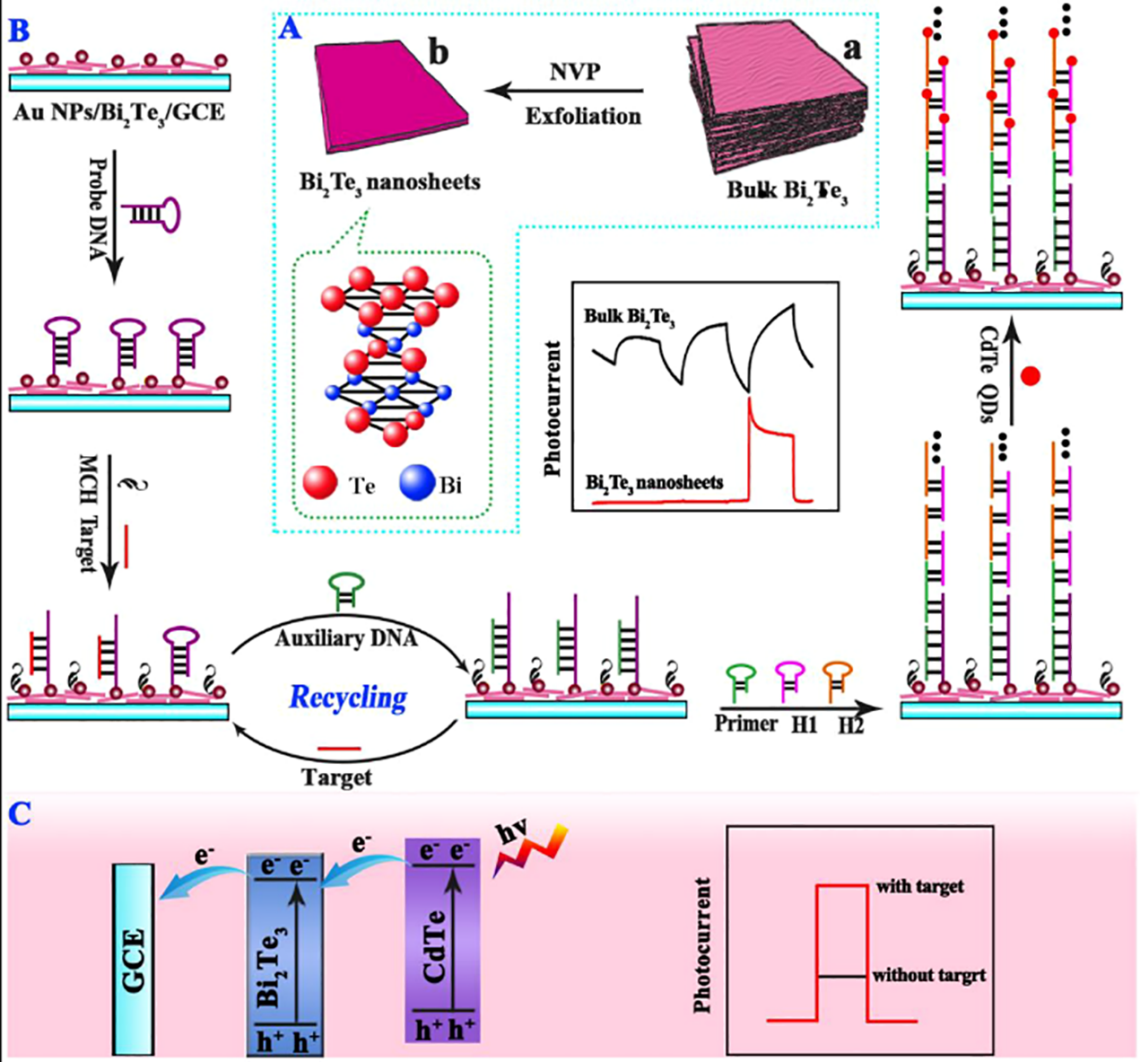
Schematic representation of (A) Bi_2_Te_3_ nanosheets’ synthesis; (B) construction of PEC biosensor, and (C) mechanism for photocurrent generation. Reprinted with permission from ref (49). Copyright 2020 American Chemical Society.

The developed biosensor (Figure 11B) was composed of a glassy carbon electrode coated with Bi_2_Te_3_ nanosheets, followed by a layer of AuNPs that was deposited into it, forming the AuNPs/Bi_2_Te_3_/GCE complex. Then, a hairpin DNA structure, named by the authors as a DNA probe, was dropped on the modified electrode surface, and due to the presence of the target the hairpin structure of the DNA probe was opened and hybridized with the target miRNA-21. Then, a second DNA probe, named by the authors as an auxiliary DNA probe, displaced the miRNA-21 to complete the amplification procedure by a strand displacement amplification process. Afterward, two more hairpin DNA structures (H1 and H2) were added to complete the hybridization chain reaction, creating long DNA tails. These DNA tails were decorated with the CdTe QDs, enhancing the photocurrent signal, due to the absorption of the UV light by CdTe QDs (Figure 11 C).

The authors concluded that the greater the concentration of miRNA-21 present in a sample, the higher the PEC signal generated by the biosensor. The proposed PEC biosensor had a LOD of 3.3 × 10^–15^ mol L^–1^, and when applied to the analysis of real samples of lysates from HeLa and MCF-7 cells, the obtained results were as expected, confirming a high expression of miRNA-21 in MCF-7 cells and a lower expression in HeLa cells. Since the developed sensor was able to detect target miRNA-21, the authors claimed that it could be used as a detection tool in clinical diagnosis.

Also in 2021, Chang et al.^126^ designed a PEC biosensor for the simultaneous detection and determination of miRNA-21 and let-7a. This photoelectrochemical biosensor consisted of DNA-functionalized CdS quantum dots, capped with MPA, for the detection of miRNA-21, and DNA-functionalized methylene blue (MB) for the detection of let-7a.

The proposed biosensor was composed by an indium tin oxide (ITO)/Au electrode divided in two parts, one for HmiR-21 linkage and the other one for Hlet-7a linkage. Only in the presence of miRNa-21 or let-7a, the configuration of HmiR-21 or Hlet-7a changed, because miRNA hybridized with the hairpin structure, to form miRNA-21@HmiR-21 or let-7a@Hlet-7a, respectively. Then, the miRNA-21@HmiR-21 complex hybridized with the DNA-functionalized CdS QDs and the let-7a@Hlet-7a complex hybridized with the DNA-functionalized MB, resulting in the immobilization of the CdS QDs and MB on the electrode surface. This process generated high PEC current for both CdS QDs and MB, which were related with the concentration of miRNA (miRNA-21 and let-7a) in the sample. According to the authors, the PEC biosensor presented LODs of 6.6 × 10^–15^ and 15.4 × 10^–15^ mol L^–1^ for miRNA-21 and let-7a, respectively.

**Figure 12:**
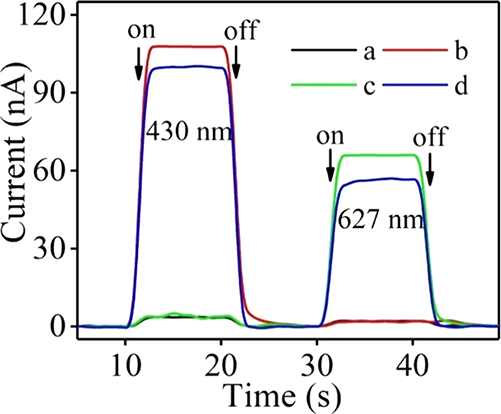
Graphical representation of the PEC signals of CdS QDs/MB-based PEC biosensor, at 430 and 627 nm, under different conditions: (a) no targets; (b) 10 pM miRNA-21; (c) 10 pM let-7a; (d) 10 pM miRNA-21 + 10 pM let-7a. Reprinted with permission from ref (126). Copyright 2021 Elsevier.

The biosensor feasibility was evaluated through specific experimental assays, in the presence or absence of the analytes, as illustrated in Figure 12. Curve a in the figure shows no significant current signal in the absence of the analytes. The addition of miRNA-21 increased the photocurrent to 108 nA (curve b), under light excitation at 430 nm, while, under 627 nm, there was no change in the PEC signal. In the presence of let-7a, the obtained result of curve c showed a higher photocurrent signal under light excitation at 627 nm (66 nA) instead of 430 nm. In the presence of miRNA-21 and let-7a, both PEC currents under 430 and 627 nm of light excitation, increased to 99.7 and 56.5 nA (curve d), respectively.

Further, to test the applicability of the proposed biosensor on the analysis of real samples, serum samples from breast cancer patients were used and the results demonstrated that the calculated concentrations of miRNA-21 and let-7a, 24.3 and 21.2 nmol/L, respectively, were in accordance with the results obtained from the conventional method, qRT-PCR. With these results, the authors concluded that the developed PEC bioassay could be used in the detection of miRNA-21 and let-7a, assisting thus in the clinical diagnosis of miRNA-related diseases.

The detection of miRNA-21 was proposed by Wen et al.^127^ in 2021, through a photoelectrochemical biosensor. The authors constructed a biosensor composed of a ternary heterostructure (SnO_2_/CdCO_3_/CdS nanomaterial) immobilized into an electrode, and a Y-type DNA structure stabilized by p-type CuS quantum dots, capped by TGA. Briefly the biosensor was assembled in this way: first, SnO_2_/CdCO_3_/CdS ternary composite materials were deposited in a glassy carbon electrode, providing a super high PEC signal. Meanwhile, double-stranded DNA was synthesized using the miRNA-21 as a model, through a cyclic reaction of polymerization to produce large amounts of output DNA. In the presence of output DNA, and after the hybridization of the “capture DNA” with p-type CuS QDs-labeled DNA, a Y-type DNA structure was created. This structure was involved in the quenching of initial PEC signal of the SnO_2_/CdCO_3_/CdS nanomaterial, as a result of the competition of photons and electron donor between p-type CuS QDs and n-type SnO_2_/CdCO_3_/CdS nanocomposite. The miRNA detection was achieved when changes in the PEC signal were verified, these changes being proportional to the concentration of miRNA-21.

In order to obtain the best experimental conditions for amplifying the photocurrent signal, the authors performed a series of optimization experiments before the construction of the sensing platform. The studied parameters were the materials dosage, the concentration of ascorbic acid (AA), the applied potential, and the dosage of Nafion. As a result of the optimization, the values found for each parameter were 10 μL of materials dosage, 0.2 mol L^–1^ AA, 0 V as the optimal applied potential, and 20 μL of Nafion.

To test the capacity of the developed biosensor for miRNA-21 detection, first a group of assays were performed by adding increasing concentrations of miRNA-21 to the sensor and measuring the obtained changes in photocurrent, being established a linear relationship between the miRNA-21 concentration and the generated photocurrent, ranging from 10 × 10^–15^ to 1 × 10^–6^ mol L^–1^. The calculated LOD value was 3.25 × 10^–15^ mol L^–1^. The study of the selectivity of the PEC biosensor was also performed, and similar sequences of miRNA were tested, namely, miRNA-203a, miRNA-155, and miRNA-141. The results showed that with these miRNAs no significant decrease in photocurrent was exhibited when compared with the blank, unlike what happened with miRNA-21. In this way, the authors concluded that the designed approach had good selectivity for the target miRNA-21 and thence can be used for the detection of miRNA-21.

### Radiative and Nonradiative Energy-Transfer-Based Sensors

Energy-transfer sensors are based on the energy transference, between the excited energy state of an electron donor to the ground state of an electron acceptor, and it can occur as a significant spectral overlap between the emission spectra of the donor and the absorption spectra of the acceptor, depending on the energy-transfer process involved.^103,129^ The transference of energy involves radiative or nonradiative excitation processes. In the nonradiative process, the photon emitted by the donor and absorbed by the acceptor is not real, since the photon is still attached to the material during the energy transfer, and it occurred when the distance between the donor and the acceptor is shorter than the wavelength, because it is a strongly distance dependent process—this is not the case during the radiative process. In a radiative process, the excitation process involves an emission of a photon from the donor, which is absorbed by the acceptor, and the effectiveness of this transference is achieved when the distance between the donor and the acceptor is larger than the wavelength.^130^

The Förster resonance energy transfer, also designated as fluorescence resonance energy transfer, is a type of nonradiative energy-transfer mechanism which results from dipole–dipole interactions between the donor and the acceptor. In this type of transfer, there is a spectral overlap, mentioned above, such that the optical transitions between the donor and the acceptor are in resonance, and the distance between the donor and the acceptor is less than 20 nm.^131^ When these conditions are verified, the energy emitted by the donor is quenched by the acceptor, resulting in the reduction of the donor’s energy emission and an increase in the acceptor energy emission. Therefore, considering the FRET mechanism and knowing that QDs are exceptional energy donors, the authors applied QDs for mainly FRET-based biosensors.^132^ In Table 3 can be found a compilation of the works present in the literature involving the biosensing of miRNA using QDS, by radiative and nonradiative energy-transfer sensors.

**Table 3:** Summarized Examples of the Use of Radiative and Nonradiative Energy-Transfer Sensors^a^

QDs	λ_max emission_ (nm)	capping	modifications and functionalization	modification method	size (nm)	target	cell line	LOD value (mol L^-1^)	ref
CdTe/CdS core–shell	580	MPA	thiolated DNA	ligand exchange		DNA		10 × 10^–15^	(13)
						miRNA-21		100 × 10^–15^	
QD605sAv	605	short 8 nucleotide DNA sequences	biotin-labeled oligonucleotides			hsa-miR-20a-5p; hsa-miR-20b-5p; hsa-miR-21–5p		1 × 10^–9^	(15)
QD655-sAv	655								
QD705-sA	705								
CdSe/ZnS	627	ODA	GSH; 50-amino-functionalized nucleic acid	ligand exchange		miRNA-141			(63)
CdTe	630	GSH	phosphorothioate-DNA		3.8	miRNA-21	HeLa; MCF-7; MDA-MB-231	4.56 × 10^–9^	(97)
ZnS		MPA-capped and Mn-doped	PDDA		5	miRNA-21		0.16 × 10^–9^	(133)
CdSe@ZnS	530	MPA	thiol-modified DNA		8	miRNA-21	HeLa	1.5 × 10^–12^	(64)
CdTe	625				5	miRNA-141	22Rv1		
CdTe	535	MPA	DNA	carbodiimide coupling (EDC/NHS)	2.23	miRNA-122		9.4 × 10^–12^	(50)
CdS_*x*_Se_1–*x*_/ZnS (core/shell)	525	oleic acid	His_6_-tag DNA	ligand exchange		miRNA-148	MCF-7; MDA-MB-231	42 × 10^–15^	(65)
	570					miRNA-21			
CdSe/ZnS core/shell	525	streptavidin			∼15	miRNA- 21	MCF-7; HEK-293; HeLa	7.2 × 10^–16^	(12)
						miRNA-221		1.6 × 10^–17^	
CdTe	631		DNA		3.7	miRNA-21	HEK-293; HeLa	10.4 × 10^–12^	(134)
CdTe	550				5	miRNA-155	MCF-7; HEK-293	12.0 × 10^–12^	(135)
CdTe	530	TGA			5	miRNA-155	SK-BR-3; MCF-7; HEK 293	0.42 × 10^–12^	(136)
CdTe	618		DNA 1–AuNPs and DNA 2–AuNPs		AuNPs, 20.8; QDs, 3.4	miRNA-21; miRNA-122			(137)
CdTe	525	mercaptoacetic acid (MAA)	DNA		2	miRNA-155	MCF-7; HEK-293	14.0 × 10^–12^	(138)
	599								
CdSe	617	multishell: CdS, CdZnS, and ZnS; silica encapsulation	DIBO; anti-AGO2 antibody	site-click coupling	20				(75)
				carbodiimide coupling (EDC/NHS)					
carboxyl-modified	525		DNA	carbodiimide coupling (EDC/NHS)		miRNA-21		36.0 × 10^–12^	(139)
	565					miRNA-20a		35.1 × 10^–12^	
	605					miRNA-155		36.5 × 10^–12^	
	650					miRNA-221		38.3 × 10^–12^	
QD605sAv	605	streptavidin	biotin-labeled oligonucleotides			miRNA-155	HL7702; MCF-7; HeLa	6.32 × 10^–17^	(140)
CdTe	542	MPA	DNA	streptavidin–biotin	2.3	miRNA-33		0.09 × 10^–9^	(141)
	630				4.1	miRNA-125b		0.02 × 10^–9^	

a TGA, thioglycolic acid; MPA, 3-mercaptopropionic acid; DNA, deoxyribonucleic acid; EDC, 1-ethyl-3-[3-(dimethylamino)propyl]carbodiimide hydrochloride; NHS, *N*-hydroxysuccinimide; miRNA, micro ribonucleic acid; GSH, l-glutathione reduced; His_6_-tag, hexahistidine; ODA, octadecylamine; AuNPs, gold nanoparticles. FePc, iron phthalocyanine; DIBO, dibenzocyclooctyne; PDDA, poly(diallyldimethylammonium chloride); QD605sAv, streptavidin-coated 605 nm-emitting quantum dots.

In 2014, Su et al.^13^ reported a turn-off QDs-based fluorescence methodology for the detection of DNA and miRNA. In this work, the authors assembled a nanosensor by a ligand-exchange substitution scheme of the MPA capping agent in core–shell CdTe/CdS QDs, by thiol-labeled single-stranded DNA (SH-DNA). The DNA-functionalized QDs allowed the differentiation of single-base mismatching and random nucleic acid sequences and, the recognition of pre-miRNA and mature miRNA. These conjugates were intended to hybridize with target nucleic acid sequences or miRNA. In this process, another nucleic acid sequence labeled with the fluorophore BHQ2 (BHQ2-labeled DNA) also hybridizes with the target analyte, quenching the QDs fluorescence, via FRET. In this work, the QDs of CdTe/CdS capped with MPA were synthesized in an aqueous phase, directly originating nanoparticles with high biocompatibility. The developed DNA-QDs nanoprobe proved to detect target DNA and miRNA-21 at concentrations 10 × 10^–15^ mol L^–1^ and 100 × 10^–15^ mol L^–1^, respectively, in 2% serum samples.

One year later, Qiu and Hildebrandt^15^ published a work describing a multiplexed “mix-and-measure” microRNA diagnostic assay using QDs and FRET between a luminescent Tb complex. Briefly, three different QDs interact with a terbium complex, to allow the detection of three different miRNAs (hsa-miR-20a-5p; hsa-miR-20b-5p; hsa-miR-21–5p) from the same sample, with a LOD value of 1 nM. Yet, these serum samples had to be diluted in a buffer solution to 10%. The main advantage of the procedure was the simplicity since it was enzyme- and amplification-free. The authors state that an ideal detection method for routine use should be single-step, sensitive, specific, rapid, reproducible, robust, storable, easy to use, versatile, and multiplexed, adding that the semiconductor nanocrystals QDs can respond to these demands. In the work, the QDs were capped with short sequences of 8 nucleotides (DNA-QD) that were complementary with a specific section of another DNA sequence conjugated with terbium (reporter DNA-Tb). In the presence of at least one of the 3 target miRNAs sequences, the DNA-QDs hybridize with other specific nucleotide sequence in the reporter DNA-Tb, originating a stable double-stranded RNA/DNA, assembled by QD-DNA, Tb-DNA, and miRNA analyte. At this stage, due to the proximity of Tb-DNA with DNA-QD, the FRET mechanism occurs, resulting in the quenching of QDs fluorescence.

In this work the authors had special attention with the design and optimization of sequence lengths and orientations of the QDs and Tb-DNA. A sequence too short in QD-DNA would result in a weak base-pairing and stacking interaction, preventing the formation of a stable double-stranded RNA/DNA FRET complex. On the other hand, a sequence too long would allow the stable hybridization between QD-DNA and Tb-DNA without miRNA analytes, which could not happen if the purpose of the method is the detection of the analytes. The selectivity of the methodology also proved to be dependent on the orientation of the QD- and Tb-DNAs. Upon the optimization assays, the number of bases for the DNAs sequences, and the orientation in QD-DNA and Tb-DNA was selected, with the condition that the tests must be carried out at temperatures between 10 and 37 °C. At lower temperatures it would form a stable QD-DNA/Tb-DNA FRET complex, with or without the miRNAs analytes; while for higher temperatures, even with the presence of miRNAs, the complexes would be unstable.

Finally, the authors demonstrated the concept by determining the miRNAs in buffer solution and in a 10% serum dilution with buffer. At room temperature, the developed homogeneous assay for 3 different miRNAs in 150 μL of sample needed 30 min of incubation and 7.5 s for total measurements (no extraction steps for miRNA were required). The QDs used in the work were Qdot 605/655/705 ITK Streptavidin Conjugate Kits.

A methodology for the fluorescent (FL) detection of miRNA-141 in serum samples was developed also in 2015 by Jou et al.,^63^ by exploiting nucleic acid-functionalized CdSe/ZnS QDs. Furthermore, the authors extended the procedure for the chemiluminescent (CL) quantification of miRNA-141, using telomerase as a means of amplification of the resulting byproducts from the detection. The procedure consisted of a two-phase sensing platform for miRNA-141. In the first phase (step 1) the QDs were functionalized, by ligand-exchange technique, with 5′-amino-functionalized nucleic acid modified at its 3′-end with BHQ2 quencher. The nucleic acid sequence capped in the QDs consisted of two important domains: domain i complementary to the miRNA-141, for FL-based detection purposes; domain ii telomerase primer, for the CL-based miRNA-141 quantification. The miRNA-141 forms a duplex with the complementary domain in the QDs. Next, the DSN cleavages the DNA strand associated with the duplex/miRNA-141, with the consequent removal of BHQ2, switching on the luminescence of the QDs and the recycling of miRNA-141, which can next bind to another probe, repeating the process, and thus, increasing the FL signal of the detection method. The DSN-mediated cleavage of domain i attached to the QDs originates a short nucleic acid single-stranded tether linked to the QDs, which is composed of domain ii plus 2–4 bases of domain i. But the presence of other miRNAs with base similarities with miRNA-141 give rise to the same detection process to some extent and hence interfere with the specificity of the miRNA-141 detection. When other miRNAs with significant base complementarity, but also with some base mismatches form a duplex with the QDs nanoprobe, a DSN-mediated partial cleavage of the structure occurs, forming domain ii with more than 8 bases, instead of 2–4 bases. This fact was exploited by the authors to implement a CL-based amplification scheme of the primary recognition phase for the selective quantification of the miRNA-141. The nucleic extended sequences are not recognized by telomerase, which allowed the high selectivity of secondary telomerase-stimulated CL quantification of miRNA-141. Telomerase, a ribonucleoprotein that is overexpressed in cancer cells, is used to promote the elongation of telomeres, by using dNTPs (building blocks of DNA). The resulting nucleic sequence after DSN activity, when miRNA-141 hybridizes with the nanoprobe, acts as primer for telomerase catalytic amplification (phase two). In phase two, the as-formed telomere chains organize into G-quadruplexes, and next, with hemin yield telomeric hemin/G-quadruplex horseradish peroxidase-mimicking DNAzyme units, which in turn catalyze the oxidation of luminol by H_2_O_2_, originating the chemiluminescence signal to be monitored. So, in the procedure, the QDs capped with domain ii and 2–4 bases of domain i were mixed with telomerase (extracted from cancer cells) and dNTPs. In the presence of hemin, the catalytic-telomeric hemin/G-quadruplexes were formed which boosted the CL signal obtained by the H_2_O_2_/luminol chemical system.

Yet, when real serum samples were tested for miRNA-141, it had an inconsistency between the results obtained by the fluorescent measurements with the ones obtained by chemiluminescent quantitative measurements of miRNA-141. This was attributed to the presence of foreign miRNAs and DNase in the samples. The interference of these structures in the samples increased the FL signal obtained during the detection phase, because these are capable of hybridizing with the QD nanoprobe and to hydrolyze the resultant hybridization, respectively, resulting overall in the release of the BHQ2 quencher and, thus, the turn-on of the QDs native fluorescence. On the other hand, the presence of DNase during the quantification procedure, decreases the concentration of the formed telomeric G-quadruplexes after miRNA-141 are recycled by the action of DSN, due to partial hydrolysis of the telomerase primer; thus, the CL signal, which is dependent on the concentration of telomeric G-quadruplexes, was smaller than what actually it should be if no interferent (DNase) was present.

One year later (2016), Ma et al.^97^ developed an interesting approach for quantification of miRNA in vitro and for direct imaging in living cells. The authors started by synthesizing gold nanoparticles and coupling thiolated DNA 1 to their surface. At the same time, DNA 2-functionalized CdTe QDs were synthesized using chimeric phosphorothioate-phosphate (ps-po) DNA as a template. A chimeric ps-po DNA molecule belongs to the class of oligonucleotides that are capable of linking to the nanostructure and, at the same time, molecular recognition of a diversity of targets. In this way, the DNA strand has two domains connected by a linker: phosphorothioate that makes the binding to the quantum dot and phosphate that is responsible for the recognition and allows the specific binding to nucleic acids and proteins, among others.

In this detection scheme, a DNA strand, named Linker, hybridizes with DNA 1–AuNPs and DNA 2–QDs, bringing these two nanostructures in proximity. These probes, comprised by a AuNP with several QDs tethered around, had the fluorescence signal quenched because of FRET from QDs to AuNPs. Next, the target miRNA strand hybridizes through branch migration with a specific nucleotide sequence in the Linker strand and initiates the release of DNA 2–QDs. Following, a DNA strand named Fuel, was necessary to also hybridize with the Linker strand, causing the release of DNA 1–AuNPs and of the miRNA target sequence, which thus is regenerated and can start another cycle with more release of DNA 2–QDs. The repetition of the cycle amplifies the fluorescence signal of QDs as more and more QDs are released from the probe. So, the miRNA target can be considered as a catalyst of the reaction, making the methodology highly sensible for target miRNAs. This scheme was applied for quantification in vitro of miRNA-21 and for the detection of the miRNA-21 in three tumor cell lines: HeLa, MCF-7, and MDA-MB-231, by confocal microscopy.

A method based on the amplification signal strategy assisted by DSN, together with phosphorescence resonance energy transfer (PRET) between PDDA-modified QDs (QDs@PDDA) and 6-carboxy-X-rhodamine (ROX)-modified miRNA sequences complementary oligonucleotide (ROX-ssDNA), was developed for the detection of miRNA-21by Yang et al.^133^ in 2017.

The detection principle of this phosphorescent probe worked as follows: QDs@PDDA are positively charged and worked as an energy donor, while ROX-ssDNA are negatively charged and worked as energy receptors. Due to this electron transference process, and the proper distance between QDs@PDDA and ROX-ssDNA, a quenching effect occurred via PRET. However, in the presence of the target, miRNA-21 hybridized first with the ROX-ssDNA, forming the DNA/miRNA-21 heteroduplexes, and then the ROX-ssDNA was cleaved by the DSN into small pieces, releasing the miRNA-21 intact for another amplification cycle. These small DNA fragments did not allow the PRET, leading to a decrease in the phosphorescence intensity, whereas the phosphorescence signal increased. The quantitative detection of miRNA-21 can be directly attained by the phosphorescence signal of QDs@PDDA.

The results obtained with the designed device showed a linear relationship between the room-temperature phosphorescence signal intensity and the miRNA-21 concentration, ranging from 0.25 × 10^–9^ to 40 × 10^–9^ mol L^–1^, the calculated LOD being 0.16 × 10^–9^ mol L^–1^. Despite the success in the detection of miRNA-21, and the capacity of the proposed method to detect other miRNAs, this work lacks assays in real samples. Nevertheless, the authors concluded that this phosphorescence probe could be used as a promising tool in miRNAs detection.

Also in 2017, a novel fluorescence probe for the dual detection of target miRNAs (miRNa-141 and miRNA-21) was proposed by Jie et al.^64^ A nuclease-aided-recycling amplification strategy was used to improve the detection of the trace levels of targets miRNA. In this way, the authors synthesized two different types of QDs (MPA-capped CdSe@ZnS and CdTe). These QDs displayed high fluorescence signals and different fluorescence emission peaks, which enabled simultaneous detection of the targets.

**Figure 13:**
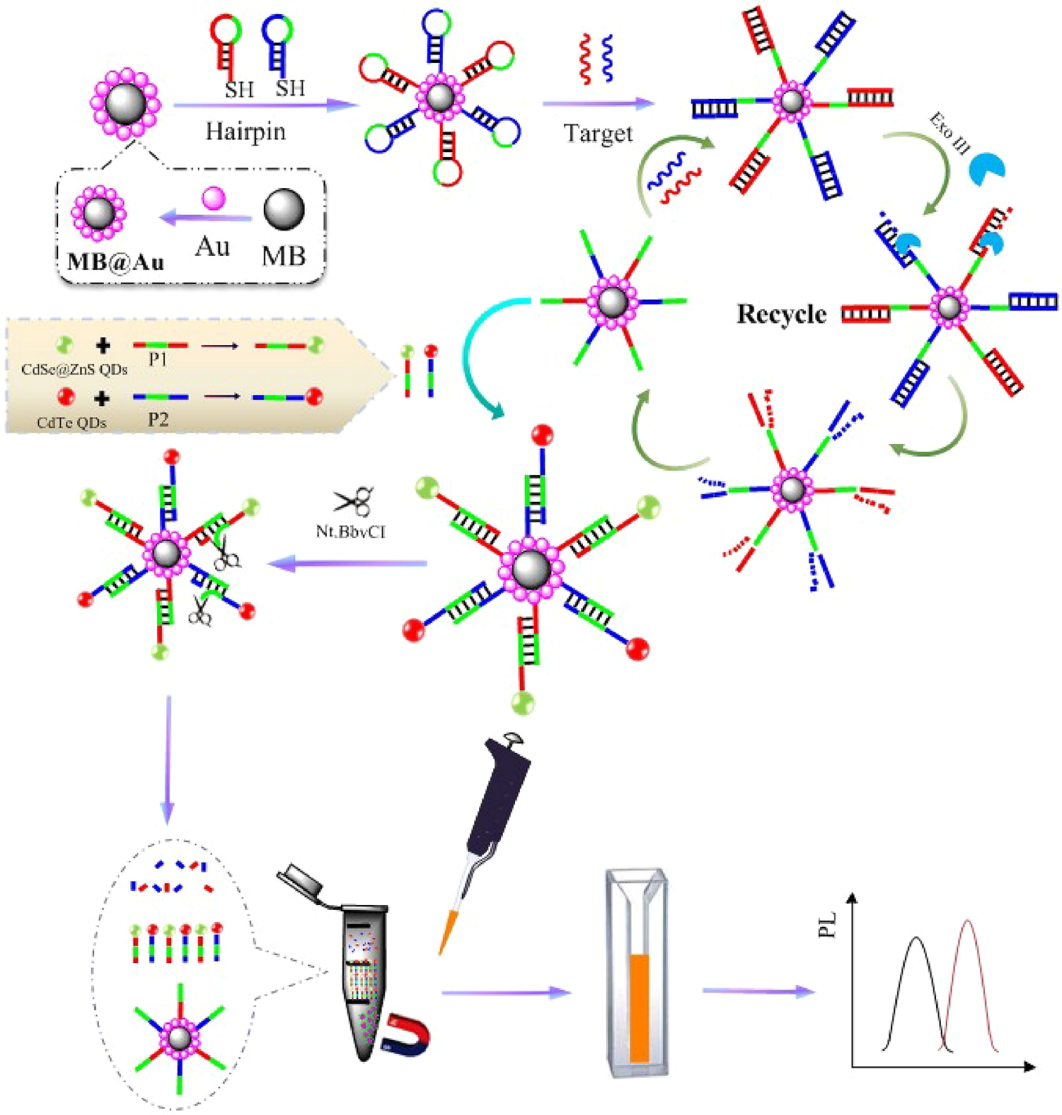
Illustration of the assembly of the proposed sensing probe for the dual detection of miRNA-141 and miRNA-21. Reprinted with permission from ref (64). Copyright 2017 Elsevier.

First, the authors synthesized AuNPs conjugated with thiol-modified magnetic beads (MBs) to form the MB@Au complex. Then, a hairpin DNA was conjugated with the MB@Au complex and, when the targets miRNA-141 and miRNA-21 were present, the hairpin structure opened to hybridize with the target, exposing the DNA strands to the action of the enzyme exonuclease III. Once it was released, the miRNA-21 or miRNA-141 was free to integrate another amplification cycle, producing many opened hairpin DNA (s1) fragments. Meanwhile, both CdSe@ZnS and CdTe QDs were functionalized with thiol-DNA P1 and thiol-DNA P2, respectively, creating the QDs probe, which was then added to the solution, for further hybridization with the s1 fragments. The Nb.BbvCI, a nicking endonuclease with the capacity to cleave only a strand of DNA in a double-stranded DNA structure, cleaved the DNA strand of the complex, allowing the release of the QDs probes to the medium. A high fluorescence intensity was obtained after the removal of QDs probes by magnetic separation. The assembly of the sensing probe described above is represented in Figure 13.

The fluorescence intensity obtained was proportional to the concentration of the miRNAs, since the bigger the fluorescence intensity, the greater the expression level of miRNA in the sample. The LOD pointed out by the authors was 1.5 × 10^–12^ mol L^–1^, for the proposed sensing platform.

The proposed method was evaluated for applicability in real samples, and HeLa cells were selected for the detection of miRNA-21, while 22Rv1 cells were chosen for the detection of miRNA-141. The test succeeded since the authors achieved the target detection and, also, the determination of the concentration of both miRNAs; the obtained results were in accordance with those reported in the literature. The authors concluded that the fabricated QD probe could be used in clinical practice for the simultaneous detection of miRNA-21 and miRNA-141, due to the results obtained with the recovery tests (97.3–104.5% for miRNA-21 and 96.3–104.2% for miRNA-141).

**Figure 14:**
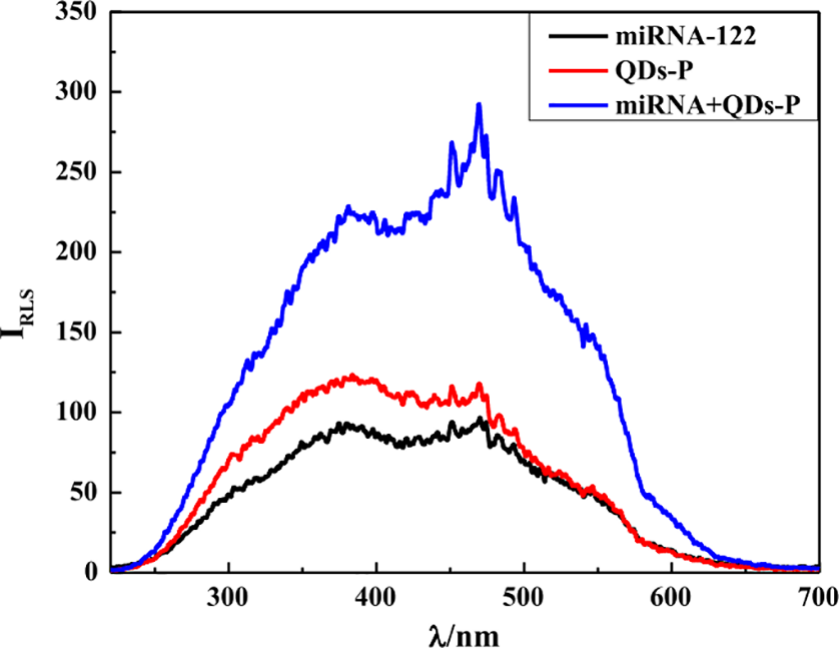
RLS spectra of the functionalized QDs (red line), miRNA-122 (black line), and miRNA+QDs-P (blue line). Reprinted with permission from from ref (50). Copyright 2017 Elsevier.

A probe for the miRNA detection based on the resonance light scattering (RLS) technique combined with CdTe QDs was developed, for the first time, in 2017 by Lv et al.^50^ On the basis of RLS theory, the authors proposed the following method: the synthesis of the MPA-capped CdTe QDs was followed by the functionalization with two cDNA-sequence probes (P1 and P2), via carbodiimide chemistry (EDC/NHS), forming QDs-P1 and QDs-P2 conjugates. Then, the conjugates were mixed, and, in the absence of the target, they did not aggregate, while, in the presence of the target, they conjugated with the miRNA-122, forming a complex of a bigger size, which enhanced the RLS intensity (Figure 14). Thus, the authors concluded that the larger the nanomaterials’ size, the greater the RLS intensity; i.e., the larger the complex area is, the stronger is the light scattering signal. The RLS intensity was proportional to the miRNA-122 concentration, which allowed the detection of the biomarker between the range 0.10 × 10^–9^ and 4.80 × 10^–9^ mol L^–1^.

After optimizing the experimental conditions, the authors applied the designed sensor in real samples (human serum spiked with increasing concentrations of the target miRNA-122) to evaluate the capacity of the sensor to detect the target. As a result, the authors obtained recoveries of 98, 87, and 110%. Despite that the proposed method does not include an amplification signal strategy, the authors concluded that the LOD obtained was lower, 9.4 × 10^–12^ mol L^–1^, than other published methods in which that strategy was applied, which constitutes an advantage. Also, the proposed device allowed a fast detection, 40 min being enough for the detection.

Wang et al.,^65^ in 2018, developed a two-step assay using an isothermal target-recycling mechanism associated with DNA-functionalized QDs for the detection of miRNA-148 and miRNA-21. The designed sensing platform was divided into two steps, the first one relating to DSN-assisted signal amplification and the second corresponding to the FRET signal generation by CdS_*x*_Se_1–*x*_/ZnS (core/shell) QDs. In step one, the target miRNA hybridized with the dye-labeled DNA probe and formed heteroduplexes, which were recognized by the DSN, and the DNA strand cleaved, leaving the miRNA intact for another round of target amplification. The cycle continued, and a lot of dye-DNA probes were released. However, the uncleaved DNA probes hybridized with DNA-functionalized QDs forming conjugates capable of generating a FRET signal. Thus, the higher the concentration of miRNA in the sample, the lower the FRET signal obtained, because only uncleaved DNA has the capacity to conjugate with QDs. The LOD pointed out by the authors was 42 × 10^–15^ mol L^–1^ to miRNA-148.

In order to study the ability of the proposed sensor to detect miRNA in biological samples, the authors chose MCF-7 and MDA-MB-231 cells and evaluated the miRNA-21 expression levels. As expected, the developed approach allowed the detection of miRNA-21 and showed that miRNA-21 is up-regulated in the selected cancer cells compared with a normal cell line (HEK 293T). Comparing the developed approach with others previously mentioned in the literature, the authors concluded that their detection scheme had advantages as it did not require washing or filtration between steps one and two and because the reaction was only two steps, allowing for the optimization of the experimental conditions individually, namely, temperature of the incubation and other parameters, in order to maximize the performance to favor either the DSN or the QDs.

A different approach for the detection and quantification of miRNA-21 and miRNA-221 was developed by Zhang et al.^12^ The authors used streptavidin-capped CdSe/ZnS core/shell QDs emitting at 529 nm. The functionalization with streptavidin allowed the conjugation of QDs with the organic fluorescent (FL) dyes Cyanine 3 (Cy3) and Texas Red, each one labeling different reporter probes. If a QD nanoparticle could allow the capping with 5–10 streptavidin molecules, and if each streptavidin had at least 3 biotin-binding sites available after its conjugation with the QD, then up to 15–30 sandwich hybrids can be formed. This means that the QDs act as signal amplifiers for the detection of miRNAs. Besides this important aspect, the QDs were also used as FRET energy donors to the FL-dyes-labeled hybrids, and these in turn allowed the selective detection of miRNA-21 and miRNA-221.

In this work, taking miRNA-21 and miRNA-221 as analytes, two circular templates respectively were designed, as well as one reverse primer for the hyperbranched rolling circle amplification (HRCA). The HRCA process was initiated in the presence of target miRNAs, Bst DNA polymerase, dNTPs, and the reverse primer. After the HRCA process occurred, several single strands of DNA products were formed. Following this, these interacted with biotinylated capture probes and the Cy3- or Texas-Red-labeled reporters, accordingly with the target analyte, to form a sandwich structure. The biotinylated capture probes were the bridge necessary for the interaction with the streptavidin QDs. Among several assays, the authors proved that the two circular templates used for the HRCA reaction do not interfere with each other, allowing thus the detection of several miRNAs analytes, simultaneously.

The authors highlighted the improved sensitivity in their methodology, due to specific hybridization between the miRNAs analytes and circular templates before HRCA reaction and consequent amplification potential as previously explained. To the overall sensitivity of the methodology contributed the efficient FRET between the QDs and the acceptors (33.7 and 35.8%, for QDs-Cy3 and QDs-Texas Red pairs, respectively), as well as the fact that each QD supports several streptavidin units and each one accepts several biotinylated capture probes, i.e., several acceptors.

Additionally, considering the specific interaction between circular templates and miRNAs analytes, HRCA products and the reporter and capture probes, streptavidin QDs, and biotinylated acceptors, altogether this contributed to the high specificity pointed out by the authors. The choice of Cy3 and Texas Red with good spectral differences also contributes to the good specificity. Finally, the methodology was able to detect and quantify miRNA-21 and miRNA-221 in MCF-7, HEK293T, and HeLa cells, by extracting first the total RNA from these.

In the same year (2018), Ma et al.^134^ proposed a methodology for dynamic sensing of miRNA in living cells, that is, time-dependent detection of miRNA. They developed a photocaged QDs-based sensor for imaging miRNA-21 in living cells, with time control. The nanosensor was based on a AuNPs core coupled to several DNA-functionalized QDs in a satellite manner (quenched via FRET by the AuNPs), through a DNA strand linker (L), with two toeholds. The linker L also supports at toehold 1 a short photocaged ssDNA containing an internal *o*-nitrobenzyl group as a photocleavable linker (PC-linker). Upon photoactivation with UV light, the PC-linker is cleaved and releases from the nanostructure the photocaged ssDNA, exposing toehold 1 for hybridization with the miRNA target. The target miRNA-21, in the presence of fuel DNA, initiates the disassembly of the AuNPs-QDs nanosensor, giving rise to emission of light from free QDs. In this process, the regenerated miRNA-21 molecules (due to action of fuel DNA) hybridize with another branch of the satellite structure, cycling the process over and over until all QDs are disassembled from the nanosensors.

This interesting scheme for nanosensor allows the user to activate the nanosensor at precisely controlled times, allowing one to collect information with time about the variations of miRNA concentrations. These can occur according to treatments and/or disease evolution.

Also in 2018, an approach for the detection of miRNA-155 was developed by Borghei and Hosseini.^135^ The authors fabricated a ratiometric fluorescence biosensor based on the green fluorescence emission of the CdTe QDs and the blue fluorescence emission of oxidized luminol (Lum_ox_).

A solution only with 3-mercaptopropionic acid-capped CdTe QDs and Lum_ox_ exhibited emission peaks at 550 and 440 nm, respectively, when excited at 350 nm. When ssDNA was added into the solution, the emission peaks were constant, and when the target miRNA-155 was present, a hybridization reaction occurred with the DNA probe, forming a DNA/miR-155 heteroduplex. This heteroduplex interacted with the CdTe QDs and triggered their aggregation, quenching their fluorescence, while the fluorescence intensity at 440 nm of Lum_ox_ remained constant. The insensitivity of Lum_ox_ allowed its use as a reference substance of the proposed assay. The quantitative detection of miRNA-155 was given by the fluorescence intensity ratios at 550 nm/440 nm, which was directly proportional to the concentration of miRNA-155, between 20 × 10^–12^ and 100 × 10^–12^ mol L^–1^, the obtained LOD being 12.0 × 10^–12^ mol L^–1^.

The authors used MCF-7 and HEK 293 cell lysates to evaluate the capacity of the designed sensor to detect miRNA-155 in real samples. As a result, they achieved the detection of the target, and when they compared with the conventional technique, namely, qRT-PCR, the results were similar, which confirmed the success of the proposed method. With this work, the authors fabricated a sensor that was simple and cheaper, in which no modifications in the as-prepared QDs were needed, and it was selective for miRNA-155, which makes it useful in the clinical diagnosis of human breast cancer.

The same team, also developed an off–on switch system for the sensing determination of the miRNA-155, by applying 90 °C to denature the DNA/RNA heteroduplex, designated by the authors as the melting temperature, in which half of the double strand begins to dissociate.^136^ The authors synthesized TGA-capped CdTe QDs, with green emission at 530 nm. Upon the addition of a ssDNA probe to QDs solution, only a slight deviation in emission was observed, while, after the heat treatment, they verified a change in the color solution, to yellow at 575 nm, and an increase in the fluorescence intensity. Then, when miRNA-155 was added, hybridization with ssDNA probe formed the DNA/miRNA-155 heteroduplex, which led to the aggregation of CdTe QDs (signal off). Consequently, applying the melting temperature, the DNA probe dissociated from miRNA-155, resulting in the disaggregation of CdTe QDs, verifying differences in the fluorescent intensities, as well as a shift from the maximum wavelength to longer wavelengths (Stoke’s shift emission), because of the increment in dipole–dipole interactions between the CdTe QDs (signal on). The quantitative detection of the miRNA can be achieved by indicating the changes in color and measuring the fluorescence intensities before and after applying the melting temperature.

The authors obtained a linear relationship between the fluorescence intensities and the miRNA-155 concentration, ranging from 10 × 10^–12^ to 100 × 10^–12^ mol L^–1^, and the calculated LOD was 0.42 × 10^–12^ mol L^–1^. The evaluation of the capacity to analyze biological samples was performed, using SK-BR-3 and MCF-7 cell lines. As a result, the authors obtained higher expression levels in the human breast carcinoma cells than in the normal cells (HEK 293). These results are in accordance with those obtained by the conventional technique qRT-PCR and also with the results previously published in the literature relative to miRNA-155. With this work, the authors proposed a new method for the quantitative detection of miRNA once it is based on the thermoresponse of CdTe QDs and can be used not only for miRNA-155 detection but also for other miRNAs.

A novel approach for tracking dual miRNAs in living cells was proposed by Ma et al.^137^ The goal of this work was to improve the identification of specific cancer cell types by the miRNA footprint, developed by the same authors years before.^97^ In this work, the intelligent sensing of a dual-miRNA profile was achieved through a QDs-based molecular computation probe with intrinsic signal amplification capacities (DNA-programmed AuNP-QD network, aka GQN).

Briefly, the GQN sensor comprised an heterobivalent DNA-functionalized QD, DNA 3–QD, by holding two different DNA–AuNPs nanostructures (DNA 1–AuNPs and DNA 2–AuNPs), through two different nucleotide sequence linkers (L1 and L2). In this structure, the QDs FL signal was quenched, due to the proximity of AuNPs, via FRET mechanism. If miRNA-21 was present, this would trigger the release of DNA 1–AuNPs from the QD, but not the release of DNA 2–AuNPs. So, the QDs FL emission remains quenched. Only if miRNA-122 was present, the DNA 2–AuNPs would be disassembled from QDs, with the consequent emission of FL signal from QDs. This means that the detection process of the two miRNAs involves amplification, computation, and output transduced in the form of fluorescence light. The authors compared the GQN sensor with a basic digital logic gate that implements AND logical conjunction: an output of 1 only happens if all inputs are 1. If any input is 0, or both, the output is 0. In this logic, 1 means QD FL signal and 0 means quenching of FL signal.

Later, in 2020, Borghei and Hosseini developed a dual-emission ratiometric fluorescence sensor, based on QDs, for miRNA-155 detection.^138^ The reported sensor comprises water-soluble green-emitting (525 nm) and orange-emitting CdTe QDs (599 nm), capped with mercaptoacetic acid (MAA or TGA).

With an off–on switch system, the sensor worked as follows: in the presence of target miRNA-155 and a double-stranded DNA (dsDNA) probe, a heteroduplex was created by the hybridization of both molecules (dsDNA/miRNA hybrid). This dsDNA/miRNA hybrid generated a strong and specific binding with green QDs, through strong interactions at their metal centers, resulting in the aggregation and fluorescence quenching of CdTe QDs. Consequently, the fluorescence intensity decreased and the off state was reached. Next, the addition of orange-QDs originated FRET, in which the fluorescence intensity at 599 nm increased, while at 525 nm decreased, resulting in a fluorescence intensity ratio at wavelengths 525 and 590 nm, this process being due to the changes in the distance between the nanoparticles when the orange-QDs are dislocated to the interspaces between the green-QDs aggregated structures.

To obtain the best sensing performance, the authors optimized some parameters, such as pH and concentration ratio of DNA to QDs. Different molar ratios of DNA:QDs were tested (10, 1, and 0.1 mol L^–1^), and after the analysis, they selected 1:1 as the best option, corresponding to the maximum effect of quenching of the fluorescence intensity of green QDs after incubating with DNA/miRNA-155 hybrid. To study the pH effect, the authors selected different pH values and verified that, under acidic conditions, the fluorescence intensity was weak, while, under slightly alkaline conditions, more specifically pH at 7.4, the difference in fluorescence intensity of green QDs, in the absence and the presence of miRNA-155, reached the maximum.

By adding increased amounts of miRNA-155, the authors evaluated the linearity of the proposed sensor and concluded that the fluorescence intensity ratio was directly proportional to the concentration of miRNA-155, at the range of 20 × 10^–12^ to 100 × 10^–12^ mol L^–1^, with the calculated LOD value of 14.0 × 10^–12^ mol L^–1^. To test the application of the reported sensor in human real samples, the MCF-7 breast cancer cell line was selected, and as a result, a high expression of miRNA-155 was detected, while with HEK 293, the cell line chosen as a negative control, the expression levels were lower. These results were in good agreement with the ones published in the literature. For the above-mentioned reasons, the authors concluded that the designed sensor can be used as a simple and rapid tool for detecting miRNA-155 in biological samples.

The immune-based sensing of circulating cell-free miRNAs (*ccf*-miRNAs) in plasma samples was accomplished by exploiting fluorescent QDs conjugated with specific antibodies (QD-Ab nanoconjugates). Shandilya et al.^75^ developed this immunoassay through two different chemistry conjugation methodologies, site-click and carbodiimide coupling. Site-click chemistry is based on the connection of an azide-modified antiargonaute protein (AGO2) antibody to dibenzocyclooctyne (DIBO)-functionalized QDs. For getting an azide-labeled anti-AGO2 antibody, the first step consists of the modification of the antibody carbohydrate domain by β-galactosidase, which creates a binding site for the attachment of GalNAz, an azide-containing sugar. In the second step, the azide-modified anti-AGO2 can be conjugated with the DIBO-functionalized QDs, creating the desired immuno-nanosensor. For the preparation of the QD-Ab nanoconjugates based on the carbodiimide conjugation chemistry, the authors used EDC and NHS for the preactivation of the carboxyl groups on the surface of QDs. Then, anti-AGO2 antibody was added to the QDs, incubating for 30 min at room temperature, leading to the formation of an amide linkage between the carboxyl groups of the QDs and amine groups of the anti-AGO2 antibody, resulting in an immuno-nanosensor.

**Figure 15:**
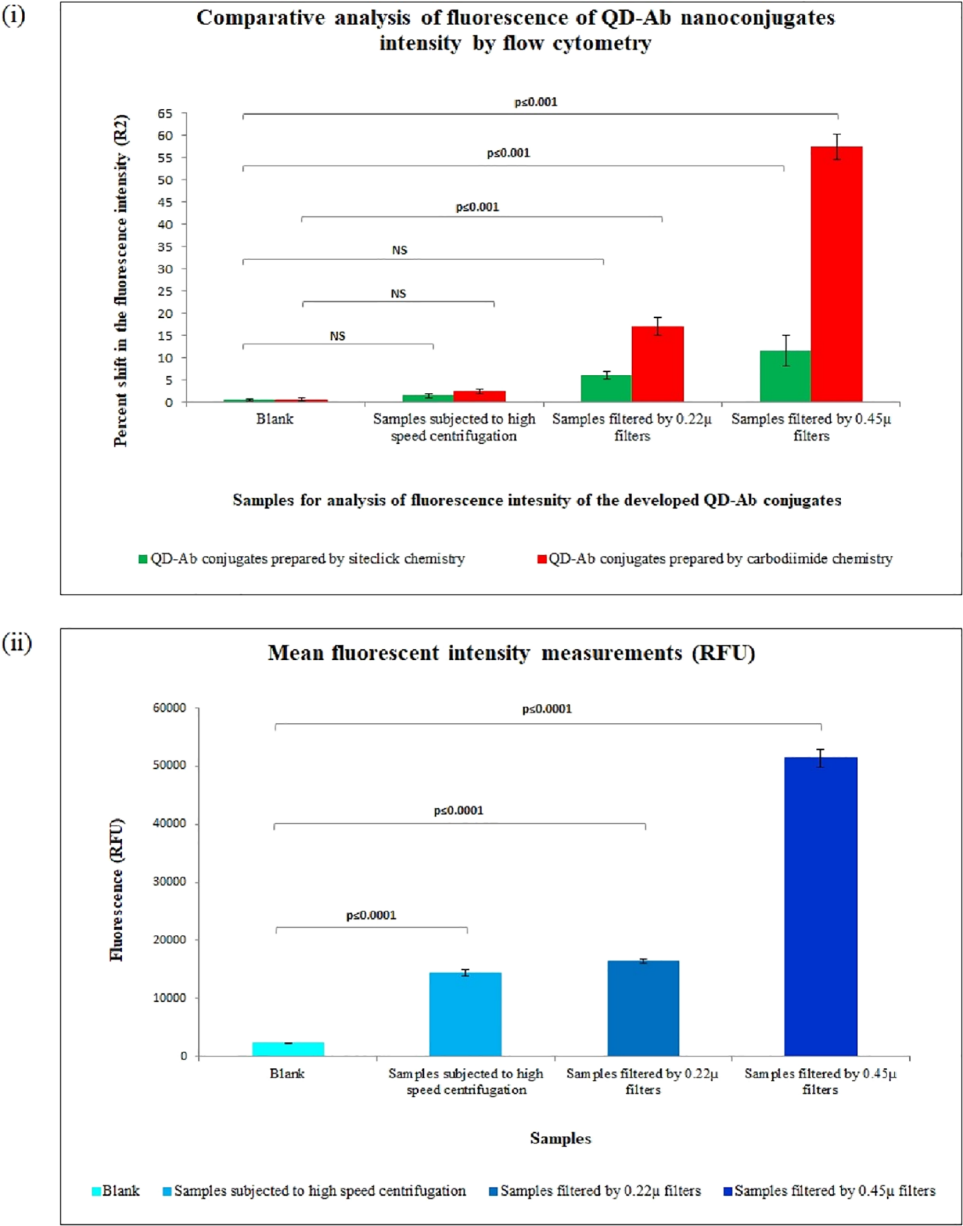
(i) Graphical representation of the results obtained through the comparative analysis of fluorescence intensity of QD-Ab nanoconjugates, synthesized by site-click and carbodiimide chemistry, by flow cytometry (mean values, *n* = 3). Bars represent mean ± SE values, and *p* ≤ 0.001 was considered statistically significant. (ii) Graphical representation of the mean fluorescence intensity measurements (*n* = 3) of the QD-Ab nanoconjugates, synthesized via carbodiimide chemistry, using fluorometry. Bars represent mean ± SE values, and *p* ≤ 0.0001 was considered statistically significant. Reprinted with permission from ref (75). Copyright 2020 Elsevier.

The detection mechanism involved a highly specific antigen–antibody immuno-binding, due to the recognition of the AGO2 proteins of *ccf*-miRNAs by the anti-AGO2 antibody-QDs conjugate. For the comparative analysis of fluorescence of both immune-nanosensors developed, the authors prepared the samples to be analyzed by flow cytometry (Figure 15). The samples were divided into four groups: samples without any type of treatment (blank); samples subjected to high-speed centrifugation; samples filtered by 0.22 μm filters; and samples filtered by 0.45 μm filters. For the graphics analysis present in Figure 15i, the authors concluded that the QD-Ab nanoconjugates prepared via carbodiimide chemistry had the best performance for the detection of AGO2 protein bound *ccf*-miRNAs, revealing high fluorescence intensities for all different samples tested, the samples being obtained after filtration by 0.45 μm filter, the ones that demonstrated a significant shift in the fluorescence intensity.

To explain the differences obtained with both developed methodologies, the authors explained that, with carbodiimide chemistry, each quantum dot particle supported two molecules of antibody, while, in the site-click approach, each QD only supported one molecule of antibody, resulting in a low fluorescence intensity response of the immune-nanosensor when prepared by site-click coupling. Thus, the authors concluded the QD-Ab nanoconjugates prepared via carbodiimide coupling presented better affinity, thus better capture and detection, for the AGO2 protein bound *ccf*-miRNA than the ones created by site-click chemistry, and for the aforementioned reasons carbodiimide methodology was elected over the other.

Since the QD-Ab nanoconjugates synthesized via carbodiimide chemistry were the ones that had the best performance for the detection of AGO2 protein bound *ccf*-miRNAs, the authors evaluated by fluorometry, the quantitative estimation of the *ccf*-miRNAs only in the samples that contained these nanoconjugates. The obtained results are graphically represented in Figure 15ii. Samples submitted to high-speed centrifugation and samples filtered by 0.22 μm filter presented similar fluorescence intensities, while samples filtered by 0.45 μm filter presented bigger fluorescence intensity. The authors’ explanation for the differences obtained between the two samples (0.22 μm filter and 0.45 μm filter) was that in the 0.22 μm filtered samples there was a removal of cell debris and microvesicles, the elimination of AGO2-bound *ccf*-miRNAs occurring, which led to lower fluorescence intensity. While in samples filtered with a 0.45 μm filter, there was the conservation of the AGO2-bound *ccf*-miRNAs and for this reason a high fluorescence intensity. So, the authors inferred that the differences in the fluorescence intensity observed between the samples filtered with 0.22 and 0.45 μm may be due to the composition of the samples.

In the same year (2020) Ye et al.^139^ proceeded to the fabrication of a microfluidic droplet chip, where four multicolor QDs were used as the fluorescence labels to detect cancer miRNAs (miRNA-20a, miRNA-21, miRNA-155, and miRNA-221). The assay was composed of a droplet chip, with a four-T-junction form, integrated with a multiplex fluorescence detection module, based on the effect of FRET between multicolor QDs and Black Hole Quencher (BHQ).

In the presence of the different target miRNAs, a hybridization reaction occurs between the QDs conjugated with a capture DNA strand, BHQ-DNA strand, and target miRNA, leading to the formation of a “sandwich” structure that provides a proper distance between the QDs and BHQ to trigger the FRET process. The addition of target miRNA caused a decrease in the fluorescence intensity, demonstrating the formation of the sandwich structure, previously mentioned, and the quenching effect of QDs by BHQ.

The quantitative determination of the four miRNAs could be achieved due to the linear relationship between the quenching efficiency of QDs and the concentration of miRNAs. With the obtained results, the authors concluded that the higher the concentration of miRNAs, the lower the fluorescence intensity of QDs-DNA conjugates, because the fluorescence was quenched by the BHQ.

An assay with healthy human serum samples was performed to evaluate the practical application of the developed detection device. In the tested samples, the targets miRNA-21, miRNA-20a, miRNA-155, and miRNA-221 were not detected, but when the samples were spiked with different concentrations of the target miRNAs, the fluorescent intensities of droplets were reduced, by increasing the miRNA concentration. The multiple detection system allowed the consumption of samples to be reduced, because only 10 nL (one droplet) was necessary to detect the analyte and improve the detection speed because it achieved the detection of 320 droplets per minute.

## Cancer Cells Bioimaging on Nucleic Acid-Functionalized Quantum Dots

Bioimaging can be considered a non-invasive method that allows the acquisition, process, and visualization of living organisms, tissues, or cells with a minimum of interference with their biological activity. In cancer cells, this type of method to visualize the cells in real-time, through the imaging probes, is important to obtain accurate information about the stage of the disease, providing an early diagnosis, and, consequently, facilitating the effectiveness of the treatment.^142^ Additionally, in the scientific research field, it provides the tools to observe bioprocesses inside cancer cells that add to the understanding of the driving mechanisms of those type of cells, opening the way for new approaches to interfere with the abnormal cellular processes in order to stop or influence the deregulated cell growth associated with cancer disease.

Nucleic acid-functionalized quantum dots are a promising tool to be used as imaging probes in cancer cells bioimaging, due to the combination of excellent optical properties of QDs with the facility of bioconjugation provided by the functionalization with nucleic acids. In the present subsection, some works involving the use of nucleic acid-functionalized QDS for cancer cells bioimaging (Table 4) were analyzed and discussed.

**Table 4:** Summarized Examples of the Use of Nucleic Acid-Functionalized Quantum Dots for Cancer Cells Bioimaging^a^

QDs	λ_max emission_ (nm)	modifications and functionalization	modification method	size (nm)	target	cell line	QY (%)	ref
Zn-doped CdTe	546	DNA		3.85 ± 0.53	MUC1	A549	>80.5	(143)
	574							
	607							
	646							
CdTe	610	DNA		4.8	Nucleolin (AS1411); mRNA	HeLa	17.8	(144)
CdTe	623	DNA	DNA-programmed hybridization chain reaction	3.5	PTK7	CCRF-CEM	18.4	(145)

a PTK7, cell surface receptor protein tyrosine kinase 7; DNA, deoxyribonucleic acid; MUC1, mucin 1; CCRF-CEM, human acute lymphoblastic leukemia cells.

One-pot hydrothermal synthesis of Zn-doped CdTe QDs and DNA-functionalized was proposed by He et al.^143^ in 2013. With this procedure, the obtained nanoparticles were photostable and had low cytotoxicity, small size, and high quantum yield. The DNA-functionalized QDs were tested for detection of MUC1, through DNA-specific recognition, as the functional domain of DNA was designed to the image of the aptamer capable of recognize mucin 1. The authors were able to successfully test the QDs, in vitro, in lung adenocarcinoma A549 cell lines, and also, in vivo in tumor-bearing nude mice. The authors opted by Zn^2+^-doped CdTe QDs for biofunctionalization because of aqueous synthesis, low cytotoxicity, high biocompatibility, high quantum yield, and small sizes of nanoparticles. Yet, the synthesis and DNA functionalization were difficult to control. Hence, in this work the authors prepared an improved method for synthesis. Very briefly, a freshly prepared NaHTe solution was added in one-pot to an oxygen-free mixture of Cd^2+^, Zn^2+^, antioxidant *N*-acetylcysteine ligand, and modified DNA, at pH 9.0. This mixture was heated to 200 °C to allow QDs growth and stabilization. With this facile one-pot hydrothermal synthesis, the DNA-functionalized Zn-doped CdTe QDs had 3.85 nm, low cytotoxicity, high quantum yield, and photostability. The assays in vitro and in vivo demonstrated excellent biocompatibility.

In 2014, Ma et al.^144^ raised the disadvantages of the several approaches for using QDs for living imaging of intracellular tumor-related markers, since the cellular uptake of QDs is achieved by endolysossomal sequestration which impairs the purpose of the QDs inside cells. The approaches involve capping QDs with endosome-disrupting polymers, delivery of QDs aided by liposome, and cell-penetrating peptides, microinjection-based delivery of QDs, and non-invasive cytosolic delivery of QDs based on biodegradable nanocomposites or special peptides. Furthermore, the signals obtained from free QDs interfere with the signals originated from interaction of QDs with target biomolecules. Ma et al. proposed a new type of DNA-functionalized QD for extra- and intracellular targeting and imaging of cancer markers. They designed a heterobivalent QD using as a DNA template a structure holding a central phosphorothioate domain and two phosphate domains at each end. The central domain was aimed at the growth of QDs, while the two different edge domains were targeted for extracellular nucleolin and intracellular mRNA. This scheme allows for the phosphorothioate DNA domain to be coupled to the Cd^2+^ ions rich surface of QDs, while the DNA phosphate domains are in a free state to react with target analytes. Aiming at nucleolin, the authors opted by AS1411 aptamer as a nucleolin-targeting motif (NTM). For surviving mRNA as target, they used a complementary 20-mer antisense DNA as mRNA-targeting motif (MTM). The MTM was additionally coupled with Cy5–oligomer and phosphorothioate linkers. During the time Cy5 was linked to the QDs probe, FRET occurred. After interaction with mRNA, Cy5 would get released and FRET was disabled, originating emission of FL signals from QDs. The purpose of the phosphorothioate linkers was minimization of enzymatic degradation of the probe.

The authors showed in their work that the micropinocytosis of the QD probe was facilitated using NTM to target cell-surface nucleolin and, consequently, facilitated the following cytosolic delivery of the QD probe for mRNA targeting inside the cell, bypassing the disadvantageous lysosomal sequestering of QDs probes which impairs sensing and imaging of intracellular targets.

A few years later, in 2016, a new approach for QD-based single-cell imaging through DNA-programmed polymerization of QDs with aptamers into linear QD-aptamers polymers was proposed by Ma et al.^145^ Thus, the cell-sensing sensitivity was enhanced by multivalent binding and multiple QDs signal amplification, as opposite to the low sensitivity when using QD-aptamers monomers. The authors’ main goal was to optimize signal amplification rather sensing of specific cancer cell markers. The assembly of the polymer followed a bottom-up approach by exploiting the hybridization chain reaction (HCR) and started with the DNA-functionalized QD monomer (M1) and the aptamer monomer (M2). The DNA-templated CdTe QDs were synthesized through phosphorothioate and phosphate chimeric DNA strands. Each monomer was constituted by the quantum dot and aptamer linked to a reactive hairpin unit (H1 or H2) through overhangs. The M1 structure was constituted by QD-H1, and the M2 structure was aptamer-H2. Next, a ssDNA initiator opens the hairpin H1 that in turn hybridizes with M2. In the last process, the parallel formation of a single-stranded region originates hybridization with another free M1. This process continues repeating until all monomers are reacted. In this work the authors were not interested in intracellular capacities of the QDs probes, but only the FL signal boost when the probes are sensing human acute lymphoblastic leukemia cells (CCRF-CEM), aiming at the cell surface receptor PTK7 (tyrosine kinase 7).

## Conclusions and Future Perspectives

miRNA is a type of RNA abnormally expressed in some diseases, namely, in cancer diseases, making it a cancer biomarker of high potential for the detection and surveillance of the disease progression. Early detection and posterior determination of those miRNAs are of supreme importance since it provides an earlier diagnosis of the disease, which the effectiveness of the cancer treatment depends on.

In the most recent years, special attention was paid to the design and construction of sensing platforms using quantum dots, due to their unique optoelectronic properties, namely, their intrinsic fluorescence and the variety of mechanisms by which their fluorescence can be quenched or enhanced, enabling several approaches aiming at analytical applications. All of the proposed methods discussed in the present work promise to be a helpful tool for miRNA detection and represent a new era with novel approaches in cancer screening procedures. When compared with the conventional techniques, such as qRT-PCR and Northern blotting, these novel sensing systems have/present the same efficacy but have extra advantages since they are simpler, faster, and of lower cost.

Considering the published scientific works thoroughly discussed in the present review, and despite the proposed detection mechanisms being pointed out by the authors as promising tools, these have not yet been transposed and approved for clinical practice, which indicates that additional research must be done to improve these methods to establish their success and advantages, to become widely available, and to guarantee a better patients’ prognosis and, consequently, to ensure the success of the treatment. Additionally, it was identified that unification of the characterization data reported in scientific papers was needed regarding the nanomaterials synthesized and utilized in biomedical applications and was particularly noticeable for the field of miRNA sensors. To make the necessary comparisons between the different approaches used for the synthesis, functionalization and application of the QDs for the cancer-related miRNA monitoring, the reported characterization techniques should be standardized, including, but not exclusively, photoluminescence emission wavelengths, hydrodynamic radius and core size, classification of the morphology of the nanoparticles, colloidal stability data (namely, ζ-potential and polydispersity index), quantum yield, and detailed description of the method used for its determination.

The successful detection of miRNAs was achieved by many authors exploiting the most diverse analytical schemes to reach the target. Several of the discussed detection schemes used a signal amplification strategy without resorting to the use of enzymes, which can be considered as an advantage, since the use of enzymes involves more controlled assays, namely, in terms of temperature. However, in most of the proposed schemes, there is a lack of comparison of the obtained results with those already published in the literature which detect the same type of biomarker since the authors only validate the results they obtain with conventional techniques.

## Vocabulary

Biocompatibility: the capacity of a material to interact with a target related with life and living processes
surface modification: designation given to QDs’ surface alterations necessary to make them appropriate for bioconjugation
surface functionalization: the process of attachment of functional molecules to the QDs, sucha as, for example, nucleic acids, antibodies, and proteins, among others
cancer-related miRNAs: 19–23 nucleotides sequences of noncoding RNA that perform regulatory functions and play a critical role in cancer’s pathology, such as in gene expression control, and some specific miRNAs are used as biomarkers since during cancer progression their levels are deregulated
hydrothermal synthesis of QDs: a bottom-up approach for production of QDs based on the reaction between the precursors when the main solvent is water
